# *Aronia melanocarpa* L. fruit peels show anti-cancer effects in preclinical models of breast carcinoma: The perspectives in the chemoprevention and therapy modulation

**DOI:** 10.3389/fonc.2024.1463656

**Published:** 2024-10-07

**Authors:** Dana Dvorska, Alena Mazurakova, Lenka Lackova, Dominika Sebova, Karol Kajo, Marek Samec, Dusan Brany, Emil Svajdlenka, Jakub Treml, Sandra Mersakova, Jan Strnadel, Marian Adamkov, Zora Lasabova, Kamil Biringer, Jan Mojzis, Dietrich Büsselberg, Karel Smejkal, Martin Kello, Peter Kubatka

**Affiliations:** ^1^ Biomedical Centre Martin, Jessenius Faculty of Medicine, Comenius University in Bratislava, Martin, Slovakia; ^2^ Department of Anatomy, Jessenius Faculty of Medicine, Comenius University in Bratislava, Martin, Slovakia; ^3^ Department of Histology and Embryology, Jessenius Faculty of Medicine, Comenius University in Bratislava, Martin, Slovakia; ^4^ Department of Pharmacology, Faculty of Medicine, P. J. Šafárik University, Košice, Slovakia; ^5^ Department of Pathology, St. Elisabeth Oncology Institute, Bratislava, Slovakia; ^6^ Department of Medical Biology, Jessenius Faculty of Medicine, Comenius University in Bratislava, Martin, Slovakia; ^7^ Department of Natural Drugs, Faculty of Pharmacy, Masaryk University, Brno, Czechia; ^8^ Department of Molecular Pharmacy, Faculty of Pharmacy, Masaryk University, Brno, Czechia; ^9^ Department of Molecular Biology and Genomics, Jessenius Faculty of Medicine in Martin, Comenius University in Bratislava, Martin, Slovakia; ^10^ Clinic of Obstetrics and Gynecology, Jessenius Faculty of Medicine, Comenius University in Bratislava, Martin, Slovakia; ^11^ Department of Physiology and Biophysics, Weill Cornell Medicine in Qatar, Qatar Foundation, Doha, Qatar

**Keywords:** *Aronia melanocarpa* L., rodent models, breast carcinoma, *in vitro* models, mechanism of action, epigenetics

## Abstract

**Introduction:**

Within oncology research, there is a high effort for new approaches to prevent and treat cancer as a life-threatening disease. Specific plant species that adapt to harsh conditions may possess unique properties that may be utilized in the management of cancer.

**Hypothesis:**

Chokeberry fruit is rich in secondary metabolites with anti-cancer activities potentially useful in cancer prevention and treatment.

**Aims of the study and Methods:**

Based on mentioned hypothesis, the main goal of our study was to evaluate the antitumor effects of dietary administered *Aronia melanocarpa* L. fruit peels (in two concentrations of 0.3 and 3% [w/w]) in the therapeutic syngeneic 4T1 mouse adenocarcinoma model, the chemopreventive model of chemically induced mammary carcinogenesis in rats, a cell antioxidant assay, and robust *in vitro* analyses using MCF-7 and MDA-MB-231 cancer cells.

**Results:**

The dominant metabolites in the *A. melanocarpa* fruit peel extract tested were phenolic derivatives classified as anthocyanins and procyanidins. In a therapeutic model, aronia significantly reduced the volume of 4T1 tumors at both higher and lower doses. In the same tumors, we noted a significant dose-dependent decrease in the mitotic activity index compared to the control. In the chemopreventive model, the expression of Bax was significantly increased by aronia at both doses. Additionally, aronia decreased Bcl-2 and VEGF levels, increasing the Bax/Bcl-2 ratio compared to the control group. The cytoplasmic expression of caspase-3 was significantly enhanced when aronia was administered at a higher dosage, in contrast to both the control group and the aronia group treated with a lower dosage. Furthermore, the higher dosage of aronia exhibited a significant reduction in the expression of the tumor stem cell marker CD133 compared to the control group. In addition, the examination of aronia`s epigenetic impact on tumor tissue through *in vivo* analyses revealed significant alterations in histone chemical modifications, specifically H3K4m3 and H3K9m3, miRNAs expression (miR155, miR210, and miR34a) and methylation status of tumor suppressor genes (*PTEN* and *TIMP3*). *In vitro* studies utilizing a methanolic extract of *A.melanocarpa* demonstrated significant anti-cancer properties in the MCF-7 and MDA-MB-231 cell lines. Various analyses, including Resazurin, cell cycle, annexin V/PI, caspase-3/7, Bcl-2, PARP, and mitochondrial membrane potential, were conducted in this regard. Additionally, the aronia extract enhanced the responsiveness to epirubicin in both cancer cell lines.

**Conclusion:**

This study is the first to analyze the antitumor effect of *A. melanocarpa* in selected models of experimental breast carcinoma *in vivo* and *in vitro*. The utilization of the antitumor effects of aronia in clinical practice is still minimal and requires precise and long-term clinical evaluations. Individualized cancer-type profiling and patient stratification are crucial for effectively implementing plant nutraceuticals within targeted anti-cancer strategies in clinical oncology.

## Graphical Abstract

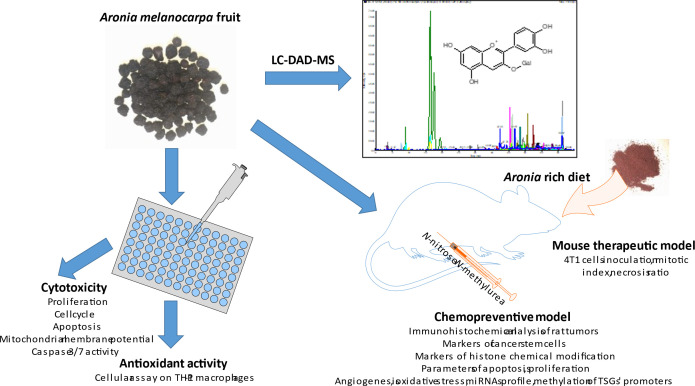

## Highlights

- In the rat chemopreventive model, aronia slightly lengthened the latency, decreased tumor volume and improved histopathological characteristics of adenocarcinomas.
- In the syngeneic 4T1 model in mice, aronia significantly decreased tumor volume of treated adenocarcinomas.
- Aronia significantly induced cancer apoptosis, and showed significant antiproliferative, anti-CSC, and antiangiogenic effects *in vivo*.
- Aronia showed significant beneficial epigenetic effects *in vivo* in the level of histone chemical modifications, miRNAs expressions, and methylation status of TSG promoters.
- Aronia demonstrated significant and complex anti-cancer properties in the MCF-7 and MDA-MB-231 cell lines and partially improved sensitivity of these cells to epirubicin.

## 1 Introduction

Breast carcinoma (BC) is currently the tumor disease most frequently diagnosed in women worldwide (1). Acquired resistance or insensitivity of cancer cells to chemotherapeutics represents a severe clinical problem of cytotoxic cancer therapies. One of the possible approaches to reducing the incidence of cancer is chemoprevention. In this context, current research emphasizes the importance of phytochemicals in reducing the risk of developing cancer as part of prevention and tries to increase the effectiveness of chemotherapeutic agents in cancer therapy. Targeting different signaling pathways to improve therapeutic outcomes by increasing the sensitivity of cancer cells and reversing resistance to currently used therapeutic methods represents an essential approach to improving the status of the clinical management of cancer (2). BC is multifactorial and is thus influenced by genetic, environmental, and lifestyle factors, including dietary preferences. Plant-based foods contain complex mixtures of phytochemicals characterized by significant biological activities, including antioxidant, immunomodulatory, anti-inflammatory, and anti-tumor effects, and also significant pro-apoptotic, anti-proliferative, anti-angiogenic, anti-stem-cell activities and epigenetic modulations of the gene expression (3–7). The mixtures of phytochemicals found in plants are potentially helpful in preventing or treating mammary carcinogenesis in humans (8).

Black chokeberry (*Aronia melanocarpa* L.) is rich in phytochemicals, which contribute to its potent antioxidative properties and various health benefits such as antimutagenic, anticancer, cardioprotective, hepatoprotective, gastroprotective, antidiabetic, anti-inflammatory, antibacterial, antiviral, radioprotective, and immunomodulatory effects (9). *A. melanocarpa* is a perennial shrub from the *Rosaceae* family, initially found in the eastern regions of North America. Aronia has also been found in Europe since the beginning of the 20^th^ century. Aronia fruits are used to prepare juices, syrups, jams, wine, or tea (9). *A. melanocarpa* is rich in proanthocyanins and anthocyanins. Besides anthocyanins, it contains significant amounts of hydroxycinnamic acids, such as chlorogenic and its isomer neochlorogenic acid, with antitumor activity *in vitro* (10). In this regard, aronia pomace using pressurized ethanol significantly decreased the viability and proliferation of HCT116 and DLD1 colon cancer cells and showed antioxidant effects (11). *A. melanocarpa* anthocyanins suppressed cell proliferation and caused cell cycle arrest in colorectal Caco cells. Additional data demonstrated that aronia anthocyanins down-regulated apoptosis *via* decreasing cytoplasmic β-catenin and the expression of related proteins in the Wnt/β-catenin signaling pathway (12). According to a study conducted by Choi et al. (13), the catechol derived from fermented aronia juice effectively suppressed the growth of BC cells and the formation of mammospheres. The inhibitory effect was observed to be dependent on the dosage of the compound. Furthermore, this catechol compound also reduced the CD44high/CD24low subpopulation, the population of cells expressing ALDH, and the genes *nanog*, *sox2*, and *oct4* associated with self-renewal. The authors concluded that catechol hinders the formation of BC stem cells by modulating the Stat3/IL-6 signaling pathway. The same authors showed that *3*-*O*-*trans*-*p*-coumaroyltormentic acid isolated from *A*. *melanocarpa* inhibits breast CSCs *via* downregulating the c-Myc protein, a CSC survival factor (14). In another study, *A*. *melanocarpa* fruit extract showed significant anti-inflammatory effects in LPS‐stimulated RAW 264.7 cells by reducing the expression of the inflammation-related biomarkers TNF-α, IL-6, and NO and the gene expression of iNOS and COX-2 (15). Thani et al. (16) have detailed the increased cytotoxic effects of gemcitabine when combined with polyphenols extracted from *A.melanocarpa*, as opposed to gemcitabine used alone, in the AsPC-1 pancreatic cancer cell line.

The antitumor properties of *A. melanocarpa* have not been studied in rodent models of BC. This comprehensive study examined the antitumor effects of orally administered A.melanocarpa in mouse 4T1 syngeneic (therapeutic) and rat chemically-induced (chemopreventive) breast adenocarcinoma models. Based on our extensive previous results with various whole plant substances analyzed in the rodent models, these goals have been formulated (17–24). The study’s subsequent objective was to evaluate the mechanism behind aronia’s anti-tumor properties by utilizing established clinical indicators of apoptosis, proliferation, angiogenesis, inflammation, cancer stem cells, and various epigenetic markers, including histone modifications, expression of oncogenic/tumor-suppressive miRNAs, and the level of methylation in crucial gene promoters linked to carcinogenesis. Furthermore, essential histopathological features of cancer cells, like the high/low-grade carcinoma ratio, mitotic activity index, and tumor necrosis ratio *in vivo* were also examined. Finally, to validate the mechanisms of the anticancer action of *A. melanocarpa* found *in vivo*, markers of proliferation, cell cycle, and apoptosis were realized using two human BC cell lines - hormone-sensitive MCF-7 and metastatic triple-negative MDA-MB-231 cells. Using the same cell lines, we analyzed the sensitization to epirubicin treatment using aronia extract.

## 2 Materials and methods

The studies received approval from the Ethical Commission of the Jessenius Faculty of Medicine at Comenius University (Protocol No. EK1860/2016) and the State Veterinary and Food Administration of the Slovak Republic (accreditation No. Ro-3239/15-221 and Ro-1640/17-221).

### 2.1 Animal models

Female Sprague-Dawley rats aged five weeks and weighing between 125–140 g (Charles River Laboratories, Sulzfeld, Germany) and female BALB/c mice aged 10 weeks and weighing between 17–19 g (Velaz, Prague, Czech Republic) were utilized in the research. The animals were acclimated to controlled vivarium conditions with specific light exposure (L/D 12: 12 h), temperature (23 ± 2°C), and relative humidity (40–60%). They were provided with a Ssniff^®^ diet (R-Z/M-Z low-phytoestrogen) (Soest, Germany) and had access to drinking water ad libitum. N-nitroso-N-methylurea (NMU, Sigma, Deisenhofen, Germany) initiated mammary gland carcinogenesis in rats through intraperitoneal injection (single dose of 50 mg/kg) on day 42 postnatal. This experimental model replicates premenopausal women at higher risk for BC development.

A therapeutic model for BC utilized a syngeneic mouse model. Subcutaneous application of 1 × 10^4^ cells/animal of 4T1 (mouse mammary adenocarcinoma) cells was performed in the abdominal mammary gland area.

The administration of aronia powder, derived from *A. melanocarpa* fruit peels (Zamio, Michalovce, Slovakia; originating from the Zemplin region), was employed as a chemopreventive substance in rats starting one week before carcinogen exposure and lasting for 14 consecutive weeks. In the allograft research, *A. melanocarpa* administration was initiated on 4T1 cell inoculation in mice and continued for 19 days. In both rat and mouse studies, *A. melanocarpa* was administered through the diet using two concentrations: a low dose of 3 g/kg - 0.3% (w/w), and a high dose of 30 g/kg - 3% (w/w). The lower dosage of aronia (0.3% in the diet) was based on our previous rich experience with dietary dosing of phytopharmaceuticals as mentioned above and by other authors (25, 26). Given the differences in pharmacokinetics and pharmacodynamics between humans and rodents, we also incorporated doses that were ten times higher in the diet for both rodent studies (3% w/w). Additionally, the elevated dosage of aronia served as an insuring measure, as there has been no prior publication on the dietary dosing of aronia in rodent BC models, and its potential oncostatic effects in rodents might have been obscured in the low-dose treated animals. The peels were processed using the “cold pelleting procedure”. The rats and mice were randomly divided into three experimental groups: a control group without chemoprevention or therapy, a group receiving chemoprevention/therapy with *A. melanocarpa* at the lower dose (aronia 0.3%), and a group receiving chemoprevention/therapy with *A. melanocarpa* at the higher dose (aronia 3%). The total number of animals used was 75 for rats and 60 for mice, with 25 animals per group for rats and 20 animals per group for mice. Starting from the fifth week post NMU administration, the rats underwent weekly palpation to assess each mammary tumor’s presence, size, and location (considered palpable if tumor diameter exceeds 0.4–0.5 cm).

Meanwhile, in mice, tumor growth (volume) was monitored a week thrice starting from the fourth-day post inoculation with 4T1 cells. Dietary intake was recorded four times for rats and twice for mice within 24-hour periods throughout the study. Subsequently, the average daily doses of *A. melanocarpa* per rat and mouse in each group were determined. Upon completion of both experiments, the animals were euthanized *via* decapitation, and mammary tumors were removed and analyzed.

### 2.2 Histopathological and immunohistochemical evaluations of rat and mouse tumors

Tissue samples from all mouse and rat adenocarcinomas were consistently subjected to formalin fixation and paraffin embedding. The rat mammary tumors were categorized based on the standardized classification criteria for mammary tumors (27). Based on an additional factor (invasive carcinoma grade), rat mammary tumors were further classified into low-grade and high-grade carcinomas. The criteria for categorization (solidization, cell atypia, mitotic activity index, necrosis) were determined based on the standard diagnostic approach for classification (28): solidization was considered if >30% of the tumor sample displayed solid growth, a high mitotic activity index of ≥10 mitoses was observed in 10 high power fields and necrosis was noted if the occurrence of comedo (not infarct) was determined (29). High-grade carcinomas were classified as tumors with two or more positive criteria, while low-grade carcinomas were classified as tumors with one or fewer positive criteria. The mitotic activity index and all tumor area/necrosis ratios were evaluated on the tumors in mice.

The most significant section of the rat mammary tumor in the paraffin block, which contained the typing characteristics and had the highest amount of vital tumor epithelial component (excluding regressive changes like extensive necrosis), was selected for immunohistochemical analysis. The markers chosen for the mechanistic study were detected using the indirect immunohistochemical method on whole paraffin sections, utilizing commercially available rat-specific antibodies (Abcam, Cambridge, MA, USA; Bioss, Woburn, MA, USA; Boster Biological Technology, Pleasanton, CA, USA; Dako, Glostrup, Denmark; GeneTex, Irvine, CA, USA; Santa Cruz Biotechnology, Paso Robles, CA, USA; Thermo Fisher Scientific, Rockford, IL, USA). Immunohistochemical staining (Autostainer Link 48/Hermes/) was performed according to the manufacturer’s recommendations. The concentration used for each primary antibody was as follows: cleaved caspase-3 1:500 (catalog no. ab2302); Bax 1:200 (sc-526); Bcl-2 1:200 (sc-492); Ki-67 1:50 (M7248 01); VEGFA 1:150 (sc-57496); VEGFR-2 1:80 (sc-6251); CD24 1:200 (gtx37755); CD44 1:200 (pa1021-2); CD133 1:150 (ab19898); ALDH1A1 1:500 (pa532127); EpCam 1:160 (ab71916); H3K4m3 1:500 (ab8580); H3K9m3 1:400 (ab8898); H4K20m3 1:300 (ab9053); and H4K16ac 1:200 (ab109463). A secondary staining technique (EnVision, Dual Link System-HRP, cat. No. K060911, Dako North America, Carpinteria, CA, USA) was employed for the visualization of primary antibodies with diaminobenzidine tetrahydrochloride as the substrate. Negative controls did not include the primary antibodies. An accurate morphometric approach was utilized to assess the expression of the immunohistochemically detected antigen. The sections were examined, and digital images were analyzed using an Olympus BX41N microscope at a magnification of ×400. The quantification of protein expression was determined by calculating the average percentage of the antigen-positive area within standard fields (0.5655 mm^2^) located in hot spot regions of tumor cells. Three hot spots were assessed per tumor sample using morphometric analysis. The morphometric analysis of digital images was conducted using QuickPHOTO MICRO software, version 3.1 (Promicra, Prague, Czech Republic). A comparison was made between treated (ARO 0.3 and ARO 3) and non-treated (control) tumor tissue samples from female rats. Sixty tumor samples per marker (900 tumor slides for 15 markers) were examined.

### 2.3 Analysis of miRNA expression

The miRVana microRNA isolation kit from Thermo Fisher Scientific and a comprehensive supplementary protocol were utilized to extract total RNA from tumor tissues. Following this, RNA quantification was carried out using a NanoDrop ND-2000 spectrophotometer from Thermo Scientific, and reverse transcription was accomplished using a TaqMan advanced miRNA cDNA Synthesis Kit from Applied Biosystems. The resulting cDNA samples were preserved at -20°C for later analysis. The tumor-suppressor miR-22, miR-34a, miR-210, and the oncogenic target miR-21 were quantitatively analyzed using the miRNA-specific TaqManTM advanced miRNA assays kit from Applied Biosystems, Life Technologies, Carlsbad, CA, USA. To ensure accurate normalization, miR-191-5p was chosen as the internal control for cDNA levels in the samples. The quantitative real-time PCR reaction was performed using an AB7500 real-time system from Applied Biosystems, Life Technologies, Carlsbad, CA, USA. All qPCR reactions were duplicated, and the average Cq values were calculated accordingly.

### 2.4 DNA isolation and bisulfite conversion

Fresh frozen tissue samples were mechanically disrupted utilizing the TissueLyser LT (Qiagen, Germany). Tissue samples weighing an average of 100 mg were loaded into 2 mL precooled tubes containing 5 mm stainless steel beads (Qiagen, Germany). The biological material was homogenized in 200 μL of lysis buffer (Qiagen, Germany) for 1 minute at an oscillation frequency of 50 Hz. The disrupted samples were then treated with 20 μL of proteinase K and incubated at 56°C. The genomic DNA extraction was performed utilizing the DNeasy blood and tissue kit from Qiagen, Germany, following the manufacturer’s instructions. Subsequently, the DNA concentration was determined using the Qubit™ 3.0 fluorometer and Qubit dsDNA BR assay kit from Thermo Fisher Scientific. The isolated DNA, with a concentration of no less than 50 ng/μL, underwent bisulfite conversion using the EpiTect bisulfite kit from Qiagen, Germany, following the additional protocol provided.

### 2.5 Determination of methylated CpG islands in the promoter regions (pyrosequencing)

Pyrosequencing was employed to analyze the methylation status of CpG islands in the promoter regions of tumor suppressor genes *TIMP3*, *PTEN*, *RASSF1A*, *PITX2*, and *ATM*. Predesigned methylation PyroMark CpG assays from Qiagen, Germany, were utilized for this purpose. The primer sequences are found in the **Supplementary Material** of the research publication (**Supplementary Table S1**). In summary, 20 ng of bisulfite-treated DNA was used in a 25 μL PCR reaction volume with the PyroMark PCR kit from Qiagen, Germany. The thermal cycling protocol included an initial denaturation step at 95°C for 15 minutes, followed by 45 cycles of amplification with denaturation at 94°C for 30 seconds, annealing at 56°C for 30 seconds, and extension at 72°C for 30 seconds. A final extension step was performed at 72°C for 10 minutes. The PCR product was then analyzed by gel electrophoresis on a 1.75% agarose gel. Subsequently, the PCR product underwent further analysis using a PyroMark Q96 ID System (Qiagen, Germany) with PyroMark Gold Q96 Reagents following the manufacturer’s instructions provided in a supplementary protocol. The data obtained from the analysis were interpreted using PyroMark Q96 software version 2.5.8 (Qiagen, Germany).

### 2.6 Preparation of aronia extracts

The powdered dried aronia peels and whole fruits were extracted using water and methanol (10 g in 100 mL) by simple maceration. The extracts were filtered, and a stream of nitrogen and lyophilization removed the solvent. Dried extracts were used for further analysis in cell models (water and methanol extracts for the Cellular Antioxidant Assay (CAA), a water extract from peels for the assays on cell lines, and LC-MS analysis of the content compounds).

### 2.7 Antioxidant activity of aronia extracts

The THP-1 cell line was obtained from the European Collection of Authenticated Cell Cultures (Salisbury, UK) and was cultured as reported previously (30, 31). The viability of THP-1 cells was measured before the antioxidant assay using the cell proliferation reagent WST-1 (Roche, Basel, Switzerland) according to the manufacturer’s manual, as reported previously (32). The antiproliferative activity of the test extracts was tested at five concentrations ranging from 10 to 0,04 μg/mL. A CAA was used to assess the antioxidant activity of the test extracts using the method of Wolfe and Liu (33) with some modifications reported previously (31) and using THP-1 cells. The solvent (DMSO) was used as a negative control (NC). Quercetin was used as a positive control at a concentration of 10 μg/mL. Statistical analyses were carried out using IBM SPSS Statistics for Windows, software version 26.0 (Armonk, NY, USA). The data were graphed as the mean ± SEM. Comparisons between groups were made using a Kruskal-Wallis test followed by pairwise comparison with Bonferroni correction.

### 2.8 Cell lines, cell cultures, and experimental design

Human BC cell lines MCF-7 (ER+, PR+, HER2−), MDA-MB-231 (ER− PR−, HER2−), and non-cancer MCF-10A (human mammary gland epithelial cells) or BJ-5ta (human dermal fibroblasts) were used in the *in vitro* experiments. BC cells were cultured in DMEM medium or RPM1 1640 medium (both Biosera, Kansas City, MO, USA), and non-cancer MCF-10A cells were cultured in DMEM F12 medium (Biosera, Kansas City, MO, USA) + supplemented with insulin, EGF- epithelial growth factor, and HC-hydrocortisone (all Sigma, Steinheim, Germany). BJ-5ta fibroblasts were maintained in a growth medium consisting of DMEM: M199 4:1 medium mixture and supplemented with Hygromycin B (0.01 mg/mL). The growth medium was supplemented with 10% FBS (Gibco, Thermo Scientific, Rockford, IL, USA) and antibiotic/antimycotic solution (Merck, Darmstadt, Germany). Cells were cultivated in an atmosphere containing 5% CO_2_ in humidified air at 37°C. Before each experiment, the cell viability was estimated by trypan blue exclusion (≥95%).

For the 3D spheroids model, the MCF-7 and MDA-MB-231 (1 x 10^4^ per well) cells were seeded into 96-well round (U) bottom Nuclon™ Sphera™ microplates (Thermo Scientific, Rockford, IL, USA) with the non-adhesive surface. The formation of spheroids was visible after 24 h and these grew for 4 days until treatment.

MCF-7 (3 × 10^5^) and MDA-MB-231 (1 × 10^5^) cells for the flow cytometry experiments were seeded in Petri dishes and cultivated for 24 h in a complete cultivation medium. The aronia extract (origin: Zamio Ltd., Michalovce, Slovakia; water extract from dried aronia peels) was added to every experimental group for 48 and 72 h before analysis.

### 2.9 Cytotoxicity assay

The resazurin assay was used to determine the cytotoxic effects of aronia extract and epirubicin (EPI) on MCF-7, MDA-MB-231, MCF-10A, and BJ-5ta cells in 2D and 3D (spheroid) models. The final aronia dilutions, ranging from 50 to 1000 µg/mL, were prepared from aronia extract solution and propylene glycol diluted in DMSO. The final EPI dilutions (in DMSO) ranged from 0.05 to 0.5 µM. The DMSO vehicle range was from v/v 0.09% (for 50 µg/mL) to 1.85% (for 1000 µg/mL). The cells were treated either after 24 hours (2D) or 4 days (3D), and the treatment lasted for 72 hours. Following the 72-hour incubation period, 10 or 15 µL of resazurin solution (Merck, Darmstadt, Germany) was added to each well, resulting in a final concentration of 40 µM. After a minimum of 1 hour of incubation, the fluorescence of the metabolic product, resorufin, was measured using an automated CytationTM 3 cell imaging multi-mode reader (Biotek, Winooski, VT, USA) with an excitation wavelength of λ 560 nm and an emission wavelength of 590 nm. The control sample’s fluorescence was considered 100%, and the results were reported as a multiple of the control. EPI autofluorescence was eliminated. All experiments were conducted in triplicate. The IC_50_ values were determined from individual treatments. The IC_12.5_ (12.5% inhibition) and IC_25_ (25% inhibition) were derived from the IC_50_ (50% inhibition) by dilution. The selectivity index (SI) was calculated by comparing the IC_50_ of the normal cell line (MCF-10A, BJ-5ta) to the IC_50_ of a cancerous cell line.

To assess the cytotoxicity of the mutual combinations of ARO and EPI, the IC_50_, IC_25_, and IC_12.5_ were selected for testing in MCF-7 and MDA-MB-231 cells. ARO and EPI were co-administered for 72 hours, and a resazurin assay was performed as previously described. The Bliss independence model as described in (34) was used to analyze the synergistic, additive or antagonistic effect of combinations. CI_Bliss_ (combinational index) = $(E_{A}+E_{B}-E_{A}E_{B})/E_{AB}$ determine the effect of drug combination as followed: A CI less than 1 indicates synergy, CI greater than 1 indicates antagonism and CI equal to 1 indicates additive/independent effect. The online application (https://sicodea.shinyapps.io/shiny/) was used to analyze data and perform an isobologram.

### 2.10 Flow cytometry analyses protocol

Floating and attached cells (MCF-7 or MDA-MB-231) were collected simultaneously 48 and 72 hours post-treatment with IC_50_ aronia extract, rinsed with PBS, suspended in PBS, and labeled (the List of flow cytometry analyses and staining) before flow cytometry examination for 15–30 minutes in the dark at room temperature as per the manufacturer’s guidelines. After a washing step, samples were resuspended in PBS, and fluorescence was detected using a FACSCalibur flow cytometer (Becton Dickinson, San Jose, CA, USA) (**Table 1**).

**Table 1:** List of flow cytometry analyses and staining.

Analyses	Staining Solution	Company
Proliferation test	CellTrace™ Yellow	Thermo Scientific, Rockford, IL, USA
	Cell Proliferation Kit (cat. no. C34567)	
Cell cycle *	10% Triton X-100	Sigma-Aldrich, Steinheim, Germany
	0.5 mg/mL ribonuclease A	
	0.025 mg/mL propidium iodide–PI	
	In 500 µL PBS	
Apoptosis	Annexin V-Alexa Fluor 647 1:100 (cat. no. A23204)	Thermo Scientific, Rockford, IL, USA
	PI (5 mg/mL) 1:500	Sigma-Aldrich
Mitochondrial membrane potential	TMRE (tetramethylrhodamine ethyl ester per chlorate) final conc. 0.1 µM	Molecular Probes, Eugene, OR, USA
Caspase 3/7 activity	CellEvent™ Caspase-3/7 Green Flow Cytometry Assay Kit (cat. no. C10427)	Thermo Scientific, Rockford, IL, USA

* After harvesting, the cell suspension was fixed in cold 70% ethanol and kept at −20°C overnight.

### 2.11 Western blot analyses

MCF-7 and MDA-MB-231 cells were exposed to IC_50_ ARO for 48 and 72 hours. To prepare protein lysates, a sonication process was employed using Laemmle lysis buffer, which consisted of 20% SDS (sodium dodecyl sulfate), glycerol, deionized H_2_O, 1M Tris/HCl (pH = 8.6), and protease and phosphatase inhibitors. The concentration of proteins was determined using a Pierce^®^ BCA protein assay kit (Thermo Scientific, Rockford, IL, USA) and measured at 570 nm using an automated Cytation™ 3 Cell Imaging Multi-Mode Reader (Biotek, Winooski, VT, USA). Electrophoresis was performed at 100 V for 3 hours on an SDS-PAA gel (10%) with a protein concentration of 40 ng. Subsequently, the proteins were transferred to a polyvinylidene difluoride (PVDF) membrane using the iBlot™ dry blotting system (Thermo Scientific, Rockford, IL, USA). The membranes underwent a blocking step for one hour at room temperature to reduce non-specific binding. This was achieved by using either 5% BSA (bovine serum albumin) from SERVA in Heidelberg, Germany, or 5% dry non-fat milk from Cell Signaling Technology^®^ in Danvers, MA, USA, along with TBS-Tween (pH = 7.4). Following blocking, the membranes were then exposed to primary antibodies (listed in the List of Western blot antibodies) overnight at 4°C. Subsequently, they were washed three times for five minutes each with TBS-Tween and incubated with a secondary antibody conjugated with horseradish peroxidase for one hour at room temperature. The chemiluminescent ECL substrate was used to detect protein expression on the washed membranes (TBS-Tween (3 × 5 min)) using the iBright™ FL1500 Imaging System (Thermo Scientific, Rockford, IL, USA). To normalize for total protein, the membranes were stained with Ponceau S solution, and the densitometry analysis of the WB bands was performed using the iBright Analysis software (Thermo Fisher Scientific, Cleveland, OH, USA) (**Table 2**).

**Table 2:** List of Western blot antibodies.

Primary antibody	Mr (kDa)	Origin	Manufacturer
PARP (46D11) mAb	116/89	Rabbit	Cell Signaling Technology, Danvers, MA, USA
Rb (4H1) mAb	110	Mouse	
Phospho-Rb (Ser807/811) (D20B12) XP mAb	110	Rabbit	
Bax (D2E11) mAb	20	Rabbit	
Anti-Bcl2 antibody [100/D5]	26	Mouse	Abcam, Cambridge, UK

Secondary antibody	Mr (kDa)	Origin	Manufacturer
Anti-rabbit IgG HRP	–	Goat	Cell Signaling Technology, Danvers, MA, USA
Anti-mouse IgG HRP	–	Goat	

### 2.12 The examinations of plant secondary metabolites in aronia extract

The methanol extract of *A. melanocarpa* fruit peels was used for the following studies. Analytical HPLC measurements were obtained on an Agilent 1260 chromatographic system (1260 Vial sampler G7129A, 1260 Quat Pump G7111B, 1260 MCT G7116A, 1260 DAD HSG7117C, Agilent Technologies, Waldbronn, Germany) with MS AB SCIEX Triple Quad 3500 system (Framingham, USA). An Agilent InfinityLab Poroshell 120 EC-C18 (4.6 × 100 mm, 2.7 μm) column with InfinityLab Poroshell 120EC-C18 (4.6 × 5 mm, 2.7 μm) guard column was used, with a gradient elution of A: MeOH and B: water with 5% HCOOH (v/v); A:B 0 min 10:90 (v/v), at the 36th min 100% A, at the 50th min 100% A. The flow rate was 0.3 mL/min, with a column block temperature of 30°C; DAD setting λ 190-600 nm. MS conditions: curtain gas N_2_ 25 L/min, temperature 450°C, gas no. 1 50 L/min, gas no. 2 40 L/min, ion spray voltage ±4500 V, scan mode *m/z* 50-1000, scan rate 1000 Da/s, solvent delay time 4 min. Compounds were identified by comparing their retention times, UV, and MS profiles with standards. The quantification was carried out by using MS (2 MRM transitions) and UV λ 510-530 nm calibration curves constructed based on measurements of the corresponding standards.

### 2.13 Statistical analyses

In animal studies, data are presented as mean ± SEM. Statistical analysis was conducted using the Kruskal–Wallis test, one-way ANOVA, Student’s t-test, and Mann–Whitney test. Tumor volume was determined using the formula: V = π × (S1)^2^ × S2/12 (where S1, S2 are tumor diameters; S1 < S2). Cell line study data are expressed as mean ± SD and analyzed with ANOVA followed by Bonferroni multiple comparisons test. ANOVA and Student–Newman–Keuls multiple comparison tests were utilized for the fluorescence assay evaluation. Significance was considered at p < 0.05. Data analyses were performed using GraphPad Prism, version 5.01 (GraphPad Software, La Jolla, CA, USA).

## 3 Results

### 3.1 Evaluation of secondary metabolites in *A. melanocarpa* fruit peel water extract

The aronia fruit peel was extracted with water. The water extract was filtered, and the water was removed by lyophilization. The lyophilized extract was analyzed using LC-MS to detect and quantify the main content compounds. The main components identified in the extract were anthocyanins, namely cyanidin arabinoside, cyanidin galactoside, cyanidin glucoside, cyanidin rutinoside, and cyanidin diglucoside, ordered from the most abundant to the least. The quantities of the anthocyanins present in the test aronia fruit peel extract are summarized in **Table 3**.

**Table 3:** Content of anthocyanins in water extract of aronia peels (in mg/g).

Anthocyanins	Content mg/g
Cyanidin galactoside	21.258 ± 1.359
Cyanidin arabinoside	8.540 ± 0.554
Unidentified	1.118 ± 0.082
Cyanidin glucoside	1.492 ± 0.1
Cyanidin rutinoside	0.311 ± 0.058
Cyanidin diglucoside	0.083 ± 0.027
In total	31.68 ± 1.99

### 3.2 Antioxidant activity of extract obtained from aronia fruit and peel

**Figure 1:**
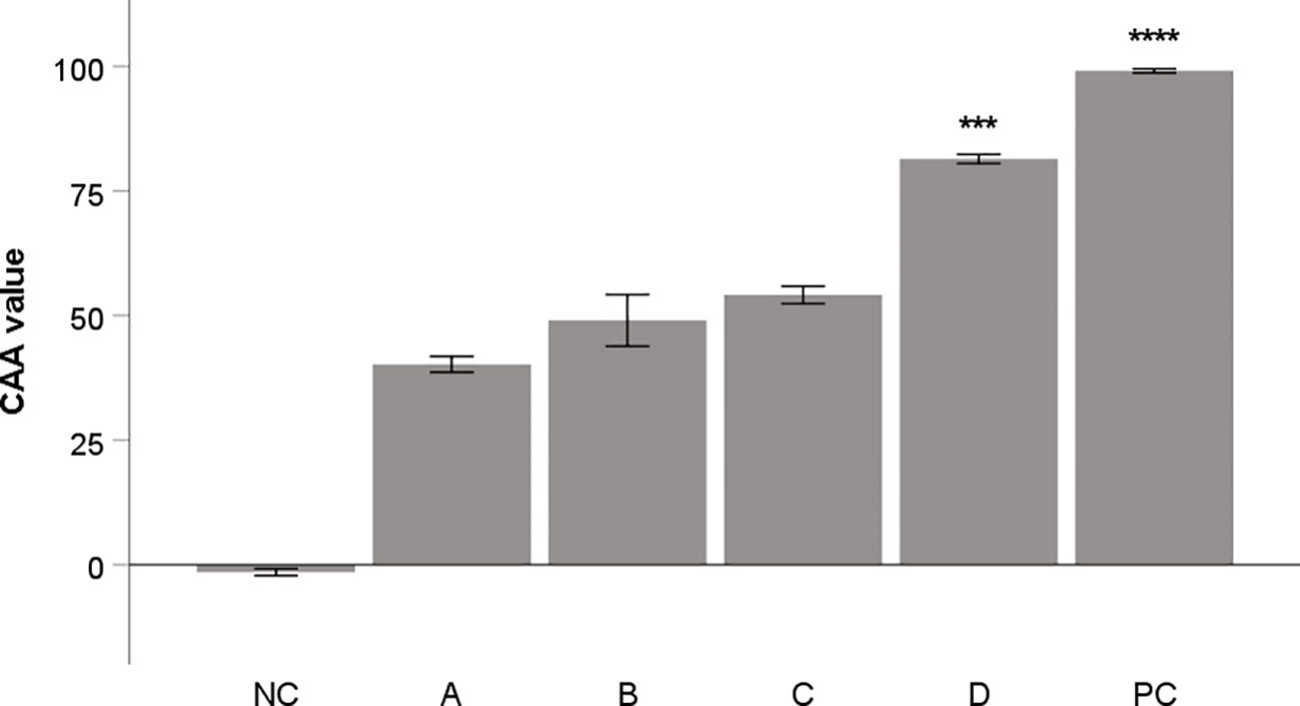
Antioxidant activity of aronia extracts (10 μg/mL) in CAA assay. Quercetin (10 μg/mL) was used as a positive control (PC). All samples were dissolved in DMSO. DMSO alone was used as negative control (NC). A: water extract of fruit, B: methanol extract of fruit, C: water extract from peel; D: methanol extract from peel. The results are expressed as the means ± SE for two independent experiments measured in triplicate (*** Indicates a significant difference in comparison with the vehicle-treated cells (NC) p < 0.001, and **** corresponds to p < 0.0001).

The methanol and water extracts from aronia dried fruit and peel were tested to evaluate their antioxidant activity in cellular assay on THP-1 macrophages. The results of these extracts were compared with flavonoid quercetin, commonly used as a standard of antioxidant activity. The results are depicted in **Figure 1**. The methanol extract from aronia peel showed the most significant activity.

### 3.3 Therapeutic mouse 4T1 model

**Figure 2:**
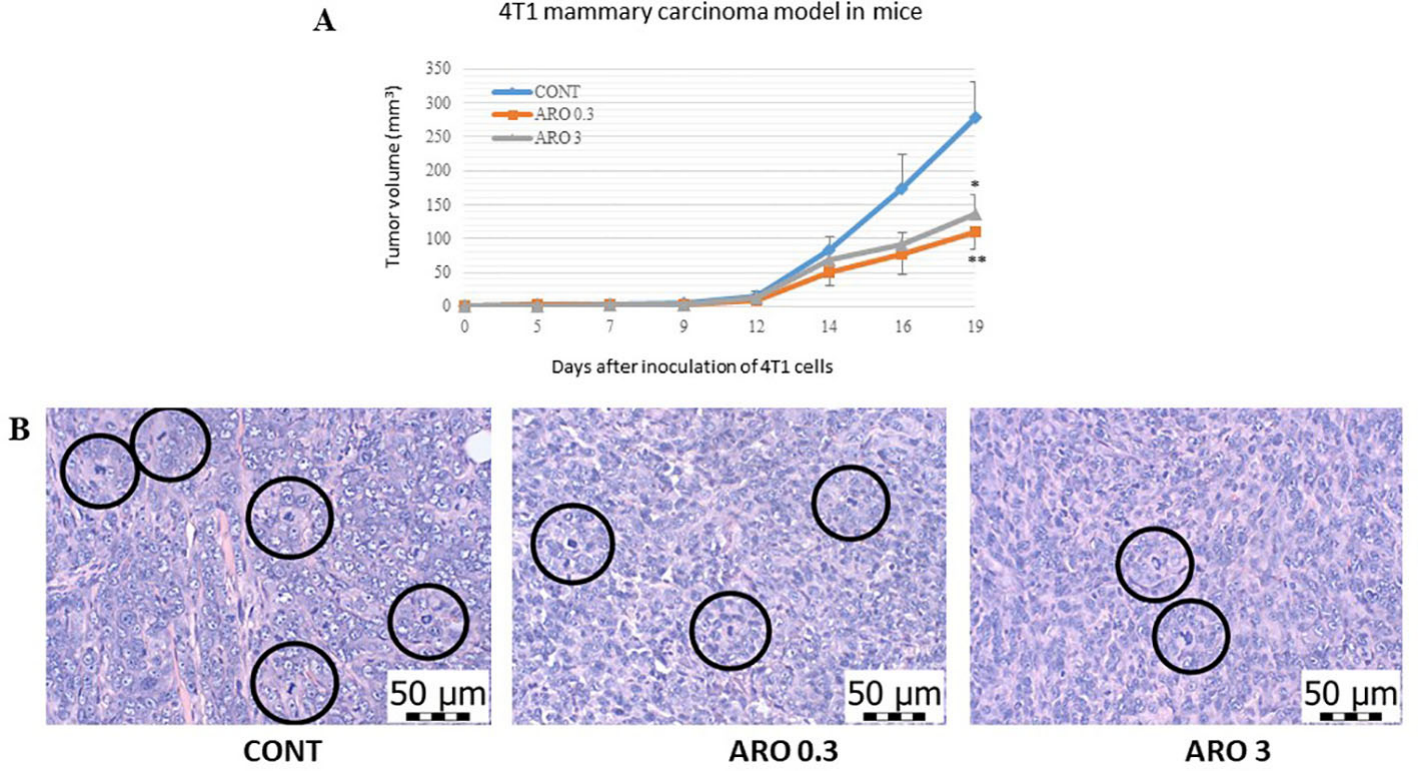
Allograft 4T1 model in mice. **(A)** The development of the volume of 4T1 mammary adenocarcinomas in mouse allograft model after *A. melanocarpa* treatment. **(B)** The mitotic activity index after treatment with *A*. *melanocarpa* extract in 4T1 tumors in Balb/c mice. The mitotic figures are highlighted in circles; H&E staining; magnification ×400. CONT—control group, ARO 0.3—a group with aronia administered at a concentration of 3 g/kg in the diet, ARO 3—a group with aronia administered at a concentration of 30 g/kg in the diet. Data are expressed as mean ± SEM. A significant difference, *P < 0.05, **P < 0.01 vs. CONT.

We observed notable effects of aronia on the reduction of 4T1 tumor volume. The administration of aronia independently led to a 60.5% decrease (P<0.01) and a 51% decrease (P<0.05) in the volume of mouse 4T1 tumors compared to the control group (KONT) (**Figure 2**). Upon histopathological analysis of the 4T1 tumor model, we observed a significant decrease in the mitotic index activity in tumor cells of the treated groups. The lower dose (ARO 0.3) resulted in a 42.5% decrease (P<0.001), while the higher dose (ARO 3) led to a 53% decrease (P<0.001) compared to the control group (CONT) (**Table 4**; **Figure 2**). Although the necrosis/whole tumor area ratio evaluation indicated reductions in the treated groups, these changes were not statistically significant due to high standard errors of the means (SEMs) in all experimental groups.

**Table 4:** Histopathological characteristics of 4T1 tumors in Balb/c mice after *A. melanocarpa* treatment.

Parameter	CONT	ARO 0.3	ARO 3
**Necrosis/whole tumor area**	7.82 ± 3.84	1.75 ± 0.46	2.04 ± 0.51
**Mitotic activity index**	20.78 ± 1.25	11.95 ± 0.91***	9.76 ± 0.95***

Data are expressed as mean ± SEM. A significant difference, ***P < 0.001 vs. CONT.

### 3.4 Chemopreventive rat mammary carcinoma model

In a chemopreventive study conducted on rats, we observed a noticeable yet statistically insignificant decrease in the ratio of poorly and well-differentiated carcinomas (high/low grade) by 34% at the lower dosage of aronia (ARO 0.3, P= 0.30) and 38.5% at the higher dosage of aronia (ARO 3, P= 0.22) compared to the control group (**Table 5**). Furthermore, the higher doses of aronia resulted in a non-significant reduction in tumor volume by 23% (P= 0.48) and an increase in tumor latency by 4.5 days (P= 0.39) compared to the control group. However, when examining other parameters of rat mammary carcinogenesis, such as tumor frequency and incidence, no significant changes were observed in the treated groups compared to the control group. The histopathological analysis of rat tumor samples revealed the presence of various types of mammary lesions, with mixed papillary/cribriform, cribriform/papillary, cribriform, and mixed cribriform/comedose carcinomas being the most frequently observed types (with the dominant type being the first in order). Additionally, sporadic lesions in rats included DCIS and inflammatory myofibroblastic tumors, as well as mixed papillary/cribriform/comedose, cribriform/papillary/comedose, and papillary/cribriform/comedose carcinomas.

**Table 5:** Effects of *A. melanocarpa* administration in chemically-induced rat mammary carcinogenesis at the end of the experiment.

Group	CONT	ARO 0.3	ARO 3
Tumor-bearing animals/all animals	17/25	17/25	20/25
Tumor frequency per group*	2.04 ± 0.45	1.96 ± 0.47	1.96 ± 0.37
Tumor latency* (days)	67.94 ± 3.83	67.94 ± 2.80	72.45 ± 3.45
Tumor incidence (%)	68	68	80
Average tumor volume* (cm^3^)	0.965 ± 0,268	0.878 ± 0.920	0.744 ± 0.163
High/low-grade carcinomas ratio	27/24 (= 1.125)	20/27 (0.741)	20/29 (0.690)

CONT, control group; ARO 0.3, a group with aronia administered at a concentration of 3 g/kg in the diet; ARO 3, a group with aronia administered at a concentration of 30 g/kg in the diet. *Data are expressed as means ± SEM.

### 3.5 Immunohistochemistry of rat tumors

#### 3.5.1 Markers of apoptosis, proliferation, and angiogenesis

**Figure 3:**
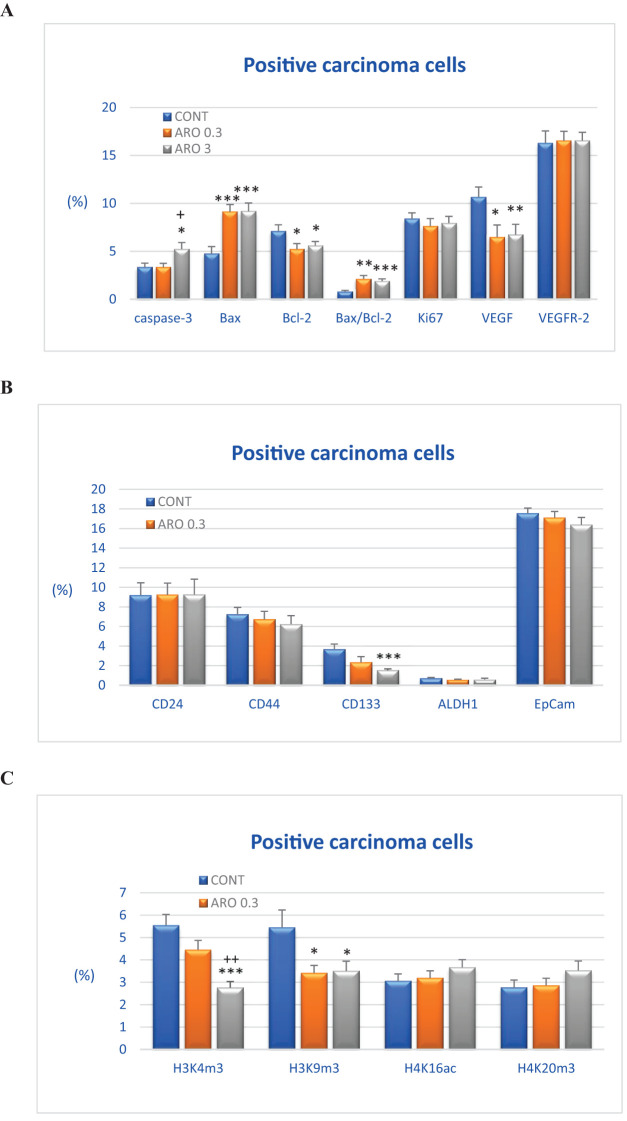
Immunohistochemical analyses of rat carcinoma cells *in vivo* after *A.melanocarpa* treatment. **(A)** Immunoexpression of cleaved caspase-3 (cytoplasmic), Bax, Bcl-2, Ki67, VEGFA, VEGFR-2, and MDA in rat tumor samples. **(B)** Immunoexpression of cancer stem cell markers in rat tumor samples. **(C)** Immunoexpression of H3K4m3, H3K9m3, H4K16ac, and H4K20m3 markers in rat tumor samples. Data are shown as mean ± SEM. A significant difference, * p < 0.05, ** *p* < 0.01, *** *p* < 0.001 vs CONT. The graphs show the protein expression, quantified as the average percentage of antigen-positive area in standard fields (0.5655 mm^2^) of hotspot areas within the tumor area. At least 60 pictures for each parameter were assessed.

The cytoplasmic expression of caspase-3 was significantly increased by 55.5% (P<0.05) with a higher dose of aronia (ARO 3) compared to the control group (CONT), and by 56.5% compared to the group with a lower dose of aronia (ARO 0.3) (P<0.05). Bax expression was significantly increased by 91.5% (ARO 0.3, P<0.001) with a lower dose of aronia, and by 92.5% (P<0.001) with a higher dose (ARO 3) compared to the control tumor samples. We observed a significant decrease in Bcl-2 expression by 27% with the lower dose of aronia (ARO 0.3, P<0.05), and by 21.5% with the higher dose of aronia (ARO 3, P<0.05) compared to the control group. The expression ratio of Bax/Bcl-2 was significantly increased by 173% (P<0.01) and 142.5% (P<0.001) with aronia, independent of the dose, compared to the control group. Aronia also significantly reduced VEGF expression by 39.5% (P<0.05) and 37% (P<0.01) compared to the control samples. The other evaluated parameters remained unchanged (**Figure 3A**).

#### 3.5.2 Markers of cancer stem cells

The assessment of tumor stem cell markers in tumor samples *in vivo* revealed a significant reduction in CD133 expression by 58.5% in the group receiving a higher dose of aronia (P<0.001) and a less pronounced decrease of 36.5% with a lower aronia dose (P=0.10) compared to the control group. In comparison to the control samples, the reductions in CD44 (by 14%), EpCam (by 6.5%), and ALDH (by 16%) expression after treatment with the higher aronia dose were not statistically significant (P>0.05) (**Figure 3B**).

#### 3.5.3 Markers of histone chemical modification

**Figure 3C** illustrates the post-translation chemical changes of histones H3 and H4 in rat carcinoma cells after being treated with aronia. The effects of aronia on these histones were dose-dependent. In comparison to the control group, the ARO 3 group showed a significant decrease of 50% in H3K4m3 levels (P<0.001), while the ARO 0.3 group exhibited a decrease of 20% (P=0.10). Both doses of aronia resulted in a reduction of H3K9m3 levels, with the ARO 0.3 group showing a decrease of 37.5% (P<0.05) and the ARO 3 group showing a decrease of 35.5% (P<0.05). Furthermore, the higher dose of aronia led to a mild increase in H4K16ac levels by 19.5% (P=0.21) and H4K20m3 levels by 28% (P=0.16) compared to the control group. However, the lower dose of aronia did not cause any significant changes in H4K16sc and H4K20m3 levels when compared to the control tumor cells *in vivo* (**Figure 3C**).

**Figure 4:**
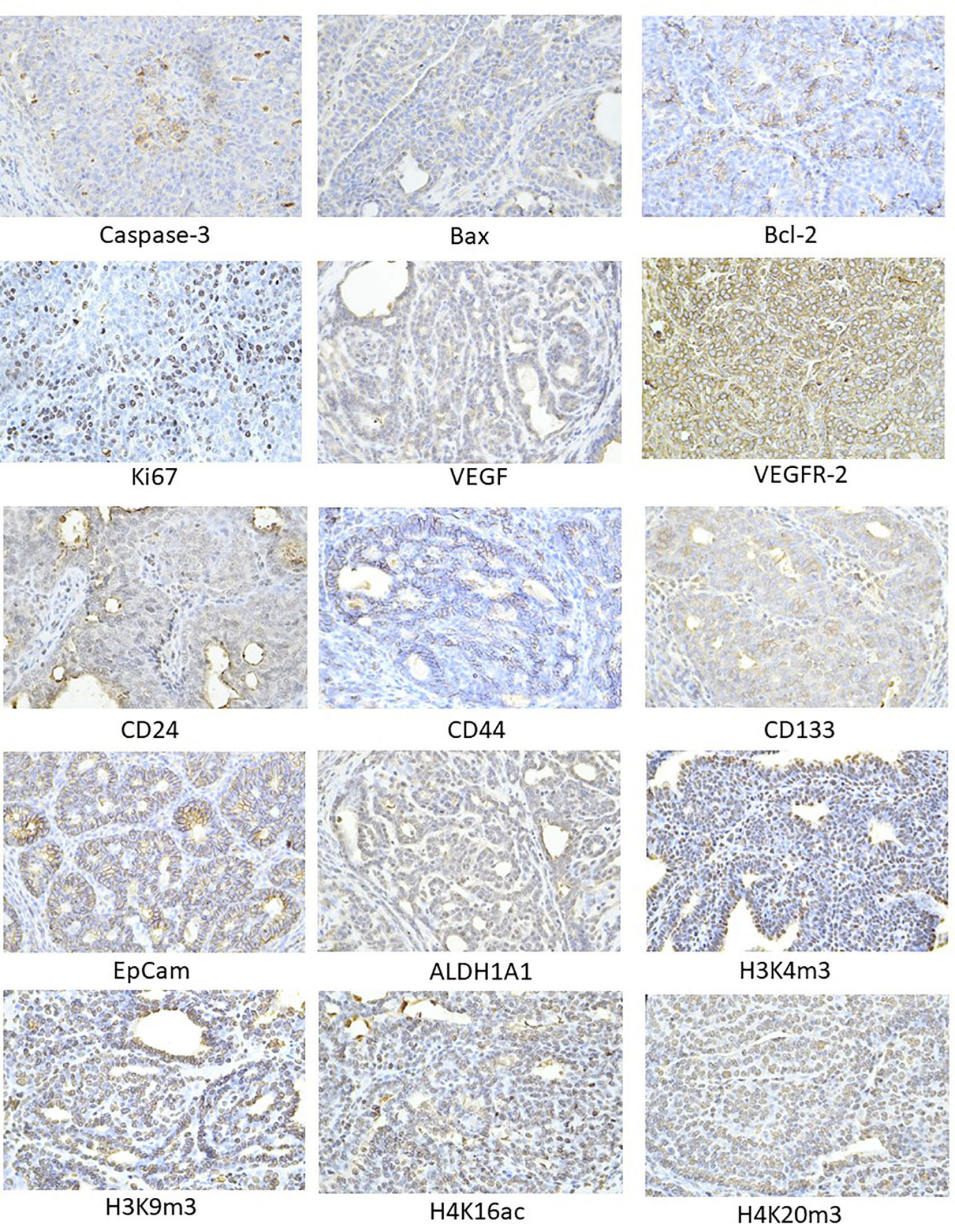
Representative images of the expression of caspase-3, Bax, Bcl-2, Ki67, VEGFA, VEGFR-2, MDA, CD24, CD44, ALDH1A1, EpCam, H3K4m3, H3K9m3, H4K20m3, H4K16ac in rat carcinoma tissue of mammary gland. For detection, polyclonal caspase-3 antibody (Bioss, Woburn, USA), polyclonal Bax and Bcl-2 antibodies (Santa Cruz Biotechnology, Paso Robles, CA, USA), monoclonal Ki67 antibody (Dako, Glostrup, Denmark), monoclonal VEGFA and VEGFR-2 antibodies (Santa Cruz Biotechnology, Paso Robles, CA, USA), polyclonal CD24 antibody (GeneTex, Irvine, CA, USA), polyclonal CD44 antibody (Boster, Pleasanton, CA, USA), polyclonal ALDH1A1 antibody (ThermoFisher, Rockford, IL, USA), polyclonal MDA, EpCAM, H3K4m, H3K9m3, and H4K20m3 antibodies (Abcam, Cambridge, MA, USA), and monoclonal H4K16ac antibody (Abcam, Cambridge, MA, USA) were applied; final magnification: ×400.

The pictures in **Figure 4** illustrate the sample expressions of cleaved caspase-3, Bax, Bcl-2, Ki67, VEGFA, VEGFR-2, CD24, CD44, CD133, ALDH1A1, EpCam, H3K4m3, H3K9m3, H4K16ac, and H4K20m3 in rat BC tissue.

### 3.6 miRNA expression in tumor tissue

**Figure 5:**
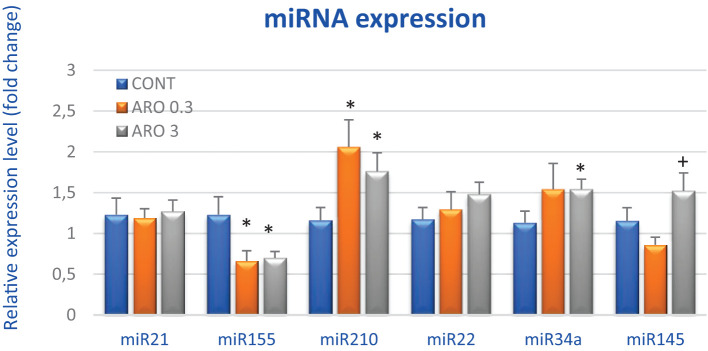
Relative miRNA expression of miR21, miR155, miR210, miR22, miR34a, and miR145 in rat mammary carcinoma samples after treatment with *A.melanocarpa*. MiR-191-5p was selected as the internal control miRNA to normalize the cDNA levels of the samples. Data are expressed as mean ± SEM. Significant difference, * *p* < 0.05 vs. CONT, + *p* < 0.05 vs. ARO 0.3.

In order to thoroughly examine the anti-cancer and epigenetic characteristics of *A. melanocarpa*, we evaluated the expression levels of six clinically validated miRNAs in rat mammary cancer tissue *in vivo* (**Figure 5**). *A. melanocarpa* notably reduced the expression of oncogenic miR155 by 46.5% (P < 0.05) at a lower dosage and by 43% (P < 0.05) at a higher dosage compared to the control group. Higher doses of aronia significantly boosted the expression of tumor-suppressive miR34a by 36.5% (P = 0.05) and slightly increased the expression of tumor-suppressive miR22 by 26.5% compared to untreated tumors. MiR210, known for its dual role in carcinogenesis as both an oncogene and a tumor-suppressor, exhibited a dose-dependent increase in cancer tissue by 77.5% (P < 0.05) and 51.5% (P < 0.05) relative to controls. Following treatment with high doses of aronia, there was an elevation in tumor-suppressive miR145 expression by 76.5% (P < 0.05) compared to the lower aronia dose. Lastly, *A. melanocarpa* did not induce any changes in the expression of oncogenic miR21 compared to the control group (**Figure 5**).

### 3.7 Promoter methylation status of tumor suppressor genes *in vivo*

**Figure 6:**
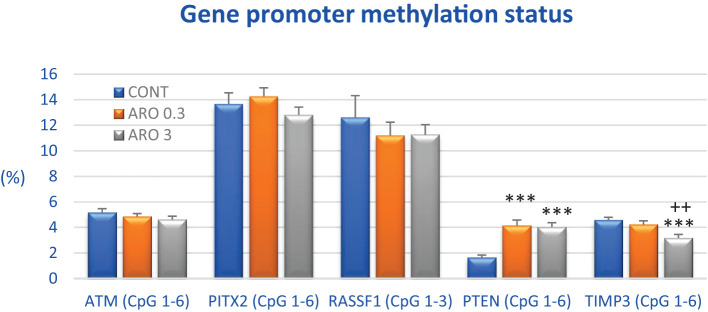
Promoter methylation status of ATM, PITX2, RASSF1A, PTEN, and TIMP3 tumor-suppressor genes in rat BC specimens after treatment with *A.melanocarpa*. The methylation level was designated using all evaluated CpG isles of the above-mentioned promoters. The brackets indicate the number of evaluated isles, i.e., *ATM* comprising six evaluated CpG sites (CpG 1–6), *PITX2* six sites (CpG 1–6), *RASSF1* three sites (CpG 1–3), *PTEN* six sites (CpG 1–6), and *TIMP3* six sites (CpG 1–6). Twenty rat BC specimens for each experimental group were analyzed. Data are shown as mean ± SEM. A significant difference, *** p < 0.001 vs. CONT group and *^++^ p <* 0.01 vs. ARO 0.3 group.

Following treatment with a higher dose of *A. melanocarpa*, there was a 30.5% decrease in total methylation of the *TIMP3* promoter compared to control samples (P < 0.001). Conversely, aronia significantly increased the methylation status of the *PTEN* promoter by 155.5% and 148% at both doses, respectively, compared with controls (P < 0.001). Evaluation of three other tumor-suppressor gene promoters revealed no significant changes, although methylation status was reduced by 11% in *RASSF1* and *ATM* genes after higher aronia dosing (**Figure 6**).

### 3.8 Physiological *in vivo* effects

During the final week of the animal studies, there were no significant differences in body weight gain and food intake following aronia administration. Rats showed good tolerance to continuous dietary aronia administration for 14 weeks. No macroscopic organ changes related to liver steatosis, hepato/splenomegaly, or gastritis were observed during the autopsy. Furthermore, there were no hematopoietic disorders or other side effects, such as fur, mucosa, or vitality abnormalities. The average daily dose of aronia per rat was 54.03 mg in the ARO 0.3 group and 536.1 mg in the ARO 3 group. For mice, the average daily doses of aronia were 9.51 mg (ARO 0.3) and 93.9 mg (ARO 3).

### 3.9 *In vitro* analyses using MCF-7 and MDA-MB-231 cell lines

#### 3.9.1 Cell viability/metabolic activity testing

An *in vitro* metabolic test was used to test the potential cytotoxic or growth-inhibitory effect of aronia extract and epirubicin (for combinational treatment) on two different BC cell lines (MCF-7, MDA-MB-231) and two different normal cell line models (MCF-10A, BJ-5ta). All cell lines were tested in the condition of monolayer growth (2D model) or as spheroids (3D model). Based on growth/metabolic activity inhibitory curves analyzed after treatment with ARO and EPI, the IC_50_ values were calculated, as seen in **Table 6**. In both BC cell lines, ARO and EPI showed concentration-dependent inhibition of metabolic activity (data not shown) in both 2D and 3D models. ARO also showed toxicity against normal cell lines, leading to an average selectivity index.

**Table 6:** Predictive IC_50_ values of ARO and EPI treatment.

	Predictive IC_50_ values ± SD (µg/mL or µM)			
	MCF-7	MDA-MB-231	MCF-10A	BJ-5ta
ARO 2D	528.0 ± 22.6	482.9 ± 36.2	713.0 ± 44.6	616.7 ± 14.6
ARO 3D	1112.4 ± 4.8	1663.9 ± 115.3	1945.2 ± 130.5	802.4 ± 64.1
Selectivity index MCF-10A (2D/3D)	1.3/1.7	1.5/1.2		
Selectivity index BJ-5ta (2D/3D)	1.2/0.7	1.3/0.5		
EPI 2D	0.43 ± 0.04	0.07 ± 0.01	0.09 ± 0.01	0.53 ± 0.02
EPI 3D	0.51 ± 0.08	1.34 ± 0.17	1.28 ± 0.21	1.49 ± 0.03

For combinational treatment (ARO+EPI), two lower concentrations were prepared from IC_50_ by half dilutions (IC_25_ and IC_12.5_) and tested for their inhibitory activity. As seen in **Table 7**, concentration-dependent inhibition on both BC cell lines is seen as expected.

**Table 7:** Resazurin metabolic test of several ARO and EPI IC_50_ dilutions in 2D and 3D models.

2D	MCF-7				MDA-MB-231			
	Concentrations							
2D	CTRL	IC_12.5_	IC_25_	IC_50_	CTRL	IC_12.5_	IC_25_	IC_50_
ARO	1.00 ± 0.00	0.96 ± 0.02	0.90 ± 0.05*	0.58 ± 0.10***	1.00 ± 0.00	0.72 ± 0.04**	0.62 ± 0.05**	0.50 ± 0.06***
EPI	1.00 ± 0.00	0.94 ± 0.10	0.83 ± 0.05*	0.54 ± 0.06***	1.00 ± 0.00	0.90 ± 0.05*	0.72 ± 0.03**	0.49 ± 0.06***
DMSO^a^	1.00 ± 0.00	1.07 ± 0.09	0.98 ± 0.07	0.92 ± 0.06	1.00 ± 0.00	1.01 ± 0.06	0.94 ± 0.06	0.91 ± 0.04
**3D**	CTRL	IC_12.5_	IC_25_	IC_50_	CTRL	IC_12.5_	IC_25_	IC_50_
ARO	1.00 ± 0.00	0.92 ± 0.03	0.72 ± 0.05**	0.61 ± 0.04**	1.00 ± 0.00	0.89 ± 0.03*	0.65 ± 0.06**	0.49 ± 0.05***
EPI	1.00 ± 0.00	0.79 ± 0.05**	0.77 ± 0.03**	0.67 ± 0.03**	1.00 ± 0.00	0.88 ± 0.04*	0.69 ± 0.05**	0.65 ± 0.04**
DMSO^a^	1.00 ± 0.00	1.01 ± 0.04	1.02 ± 0.04	0.93 ± 0.05	1.00 ± 0.00	1.04 ± 0.02	1.01 ± 0.06	0.91 ± 0.04

a DMSO was used as v/v % equivalent of IC; *p > 0.05, **p > 0.01, *** p > 0.001 vs untreated control. Data are presented as a fold of the control.

#### 3.9.2 Antiproliferative activity of aronia extract

**Figure 7:**
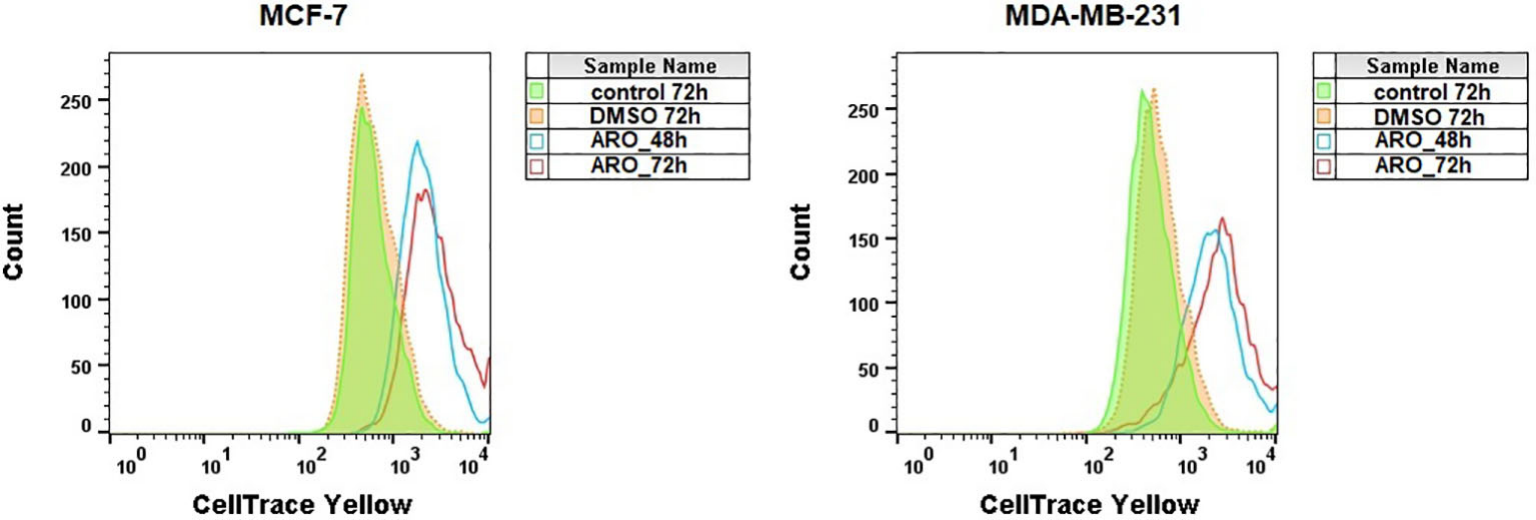
Analysis of MCF-7 and MDA-MB-231 cells proliferation stained with CellTrace™ Yellow after ARO IC_50_ treatment. Representative histograms.

The cell-permeable fluorescence dye CellTrace™ Yellow was used to analyze the proliferation of cells after ARO treatment. **Figure 7** illustrates that treatment with ARO IC_50_ effectively suppressed the growth of both cell lines at 48 and 72 hours, in contrast to the normal proliferation of DMSO/untreated cells at 72 hours.

#### 3.9.3 Cell cycle analyses

**Figure 8:**
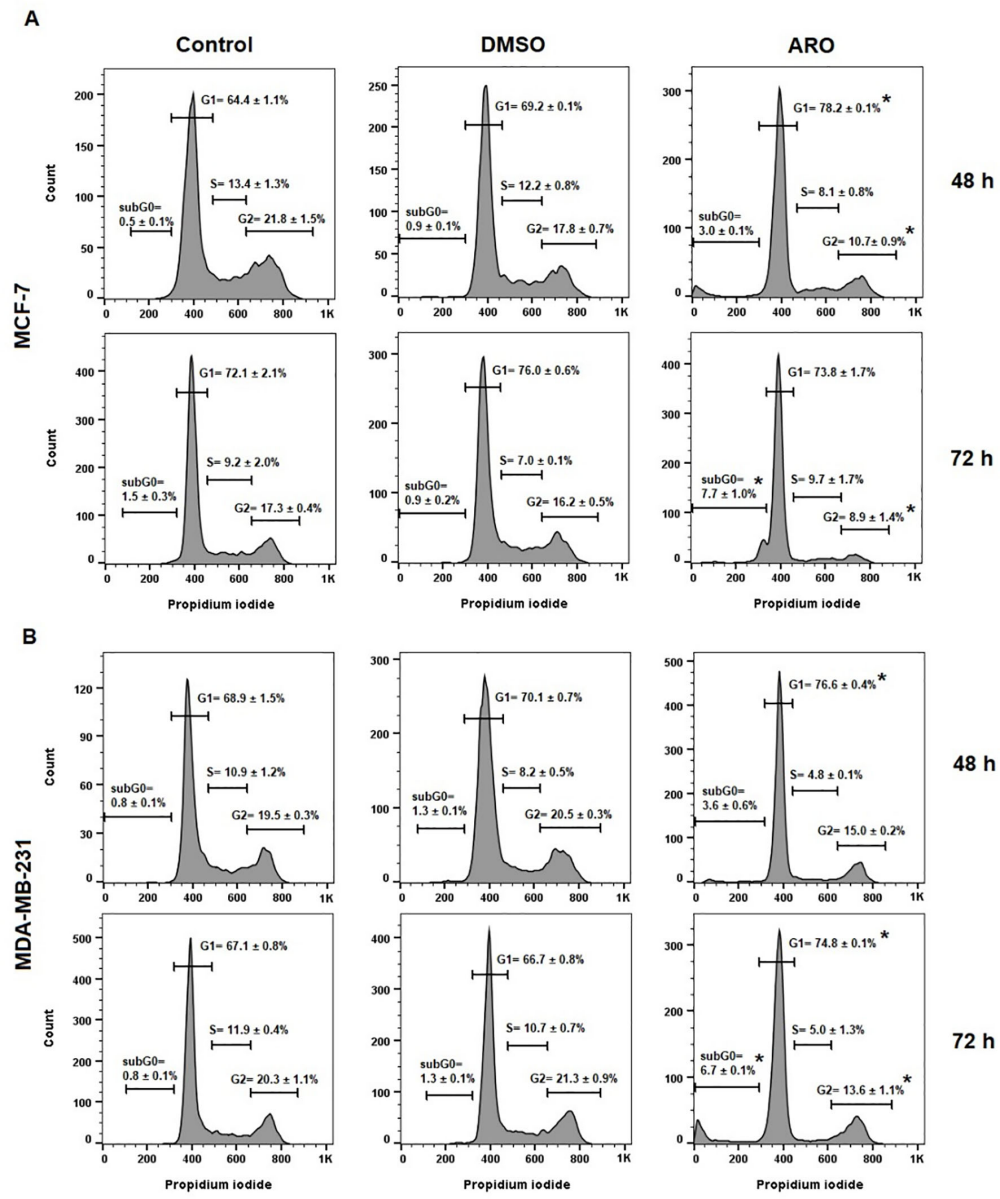
Cell cycle distribution of MCF-7 **(A)** and MDA-MB-231 **(B)** cells after 48 and 72 h of ARO IC_50_ treatment. Statistical significance: *p > 0.05 vs untreated control. Representative histograms with average data ± SD.

The effects of ARO IC_50_ treatment on the cell cycle were examined in cancer cell lines. Both BC cell lines showed G1 cell cycle arrest at 48 h following ARO treatment, with or without continuation up to 72 h (**Figure 8**). Simultaneously, there was a concomitant decrease in the number of cells in other cell cycle phases and an increase in the apoptotic subpopulation (subG0) at 72 h. SubG0 represented a subpopulation with fragmented DNA.

#### 3.9.4 Apoptosis analyses

**Figure 9:**
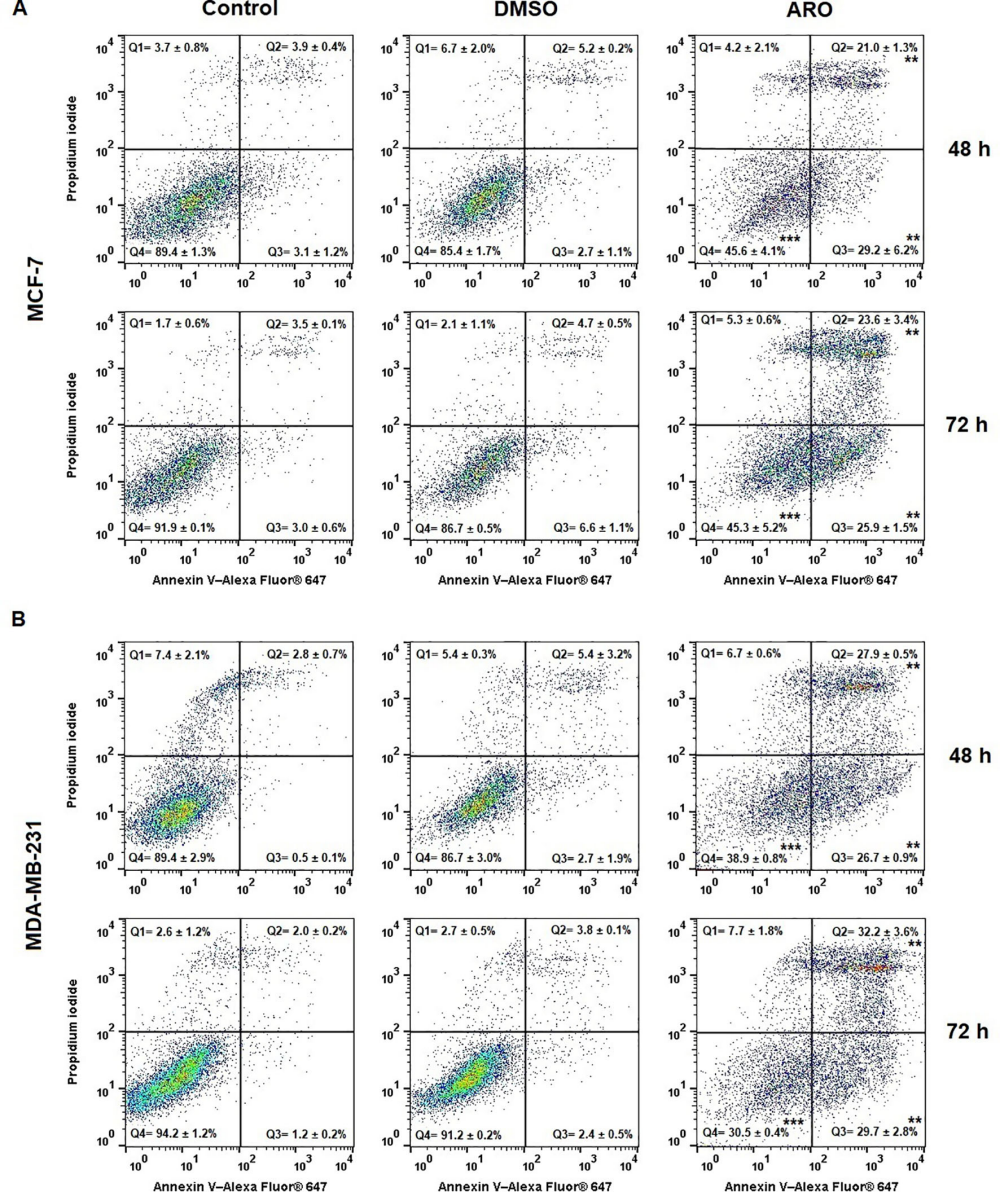
Occurrence of apoptosis in populations of MCF-7 **(A)** and MDA-MB-231 **(B)** cells after 48 and 72 h of ARO IC_50_ treatment. Statistical significance: **p > 0.01, ***p > 0.001 vs untreated control. Representative dot plots with average data ± SD. Legend: Q1 (Death), Q2 (Late apoptotic), Q3 (Early apoptotic), Q4 (Live).

The externalization of phosphatidyl serine (PS) is an early indicator of cell death, as demonstrated by Annexin V/PI staining, which categorizes the population into four groups: Q4= Live (An-PI-), Q3= Early apoptotic (An+PI-), Q2= Late apoptotic (An+PI+), and Q1= Dying cells (An-PI+). **Figure 9** shows that both cell lines ARO showed a gradual increase in the number of early and late apoptotic cells with externalized PS over time. By the 72-hour post-treatment, the early apoptotic cells transitioned slightly towards the late apoptotic phase, contributing to the dying cell population.

#### 3.9.5 Mitochondrial potential changes and analyses of caspases activity

**Figure 10:**
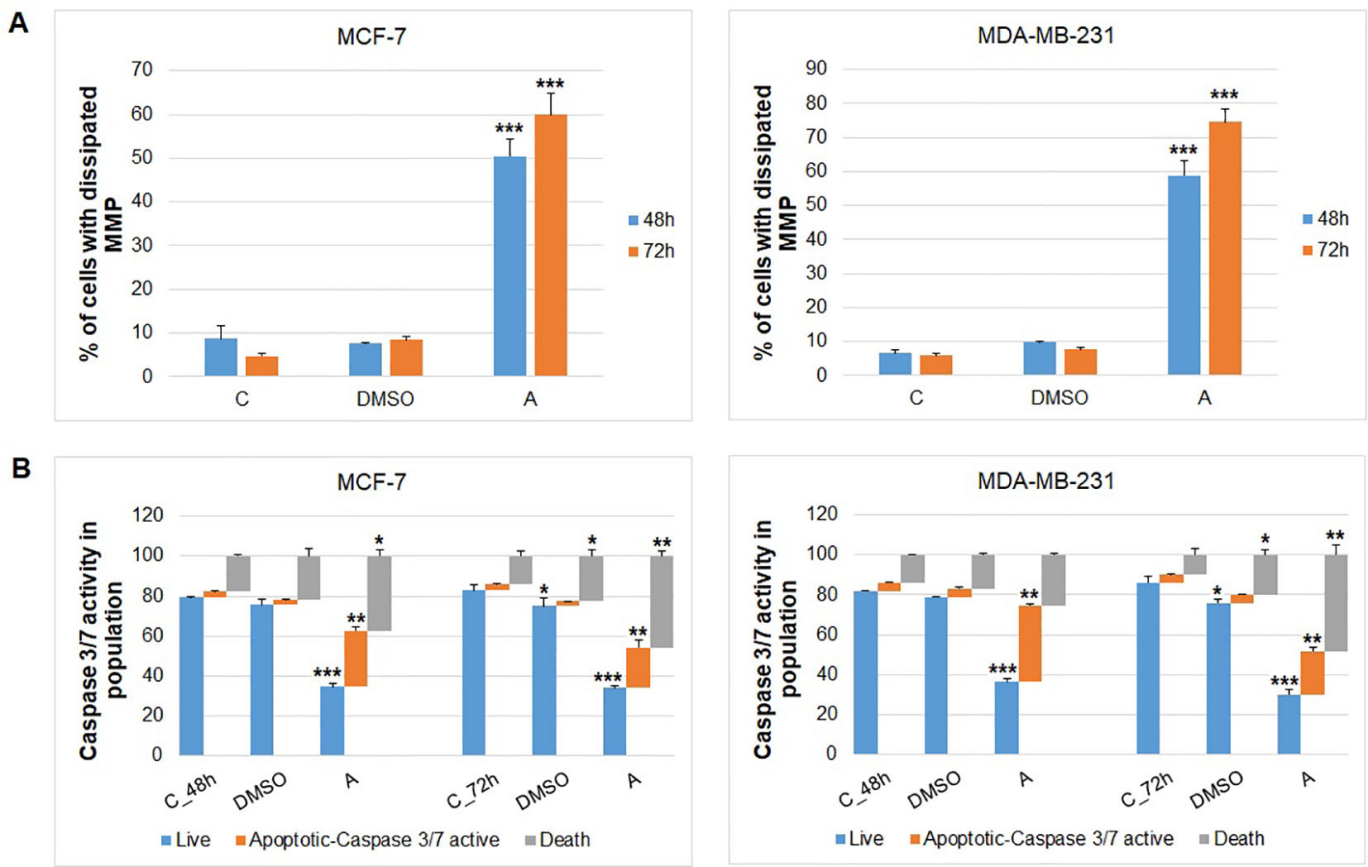
Mitochondrial membrane potential dissipation **(A)** and caspase 3/7 activity **(B)** in MCF-7 and MDA-MB-231 cells after 48 and 72 h of ARO IC_50_ treatment. Statistical significance: *p > 0.05, **p > 0.01, ***p > 0.001 vs untreated control.

The potential pro-apoptotic effects of ARO treatment were examined in MCF-7 and MDA-MB-231 cells, focusing on changes in mitochondrial function and the initiation-execution of apoptotic mechanisms. **Figure 10A** demonstrates that ARO treatment resulted in the dissipation of mitochondrial membrane potential at all time points tested in both cell lines. Additionally, the caspases activity test revealed that ARO treatment triggered the activation of executioner caspases 3/7, reaching their peak activity at 48 hours in both cell lines (**Figure 10B**). Furthermore, ARO treatment increased the population of cells with damaged membranes, indicating cell death.

#### 3.9.6 Western blot analyses of protein expression

**Figure 11:**
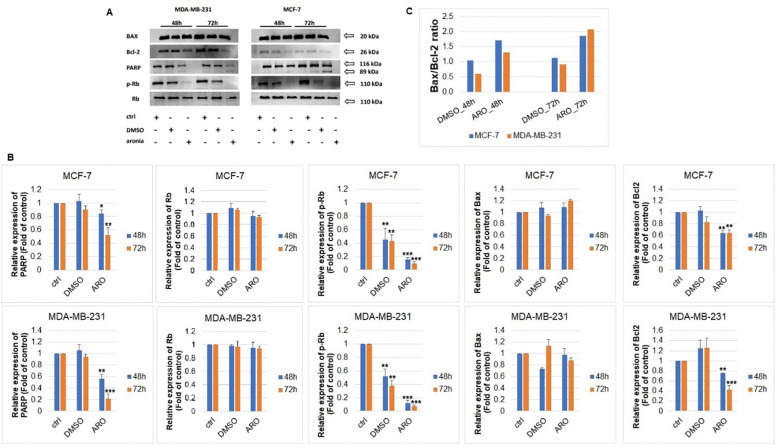
Western blot analyses of apoptosis-associated proteins **(A)** after ARO IC_50_ treatment of MCF-7 and MDA-MB-231 cells include densitometry band analyses **(B)** and Bax/Bcl-2 ratio **(C)**. Statistical significance: *p > 0.05, **p > 0.01, ***p > 0.001 vs untreated control.

The analysis of PARP, p-Rb/Rb, Bax, and Bcl-2 protein expressions through western blot was carried out following ARO IC50 treatment on MCF-7 and MDA-MB-231 cells. **Figures 11A, B** illustrates that the ARO treatment resulted in the cleavage of PARP due to caspase 3/7 activity. Additionally, a reduction in phosphorylated Rb, a cell cycle regulator in the G1/S transition, was noted after ARO treatment in both cell lines. Total Rb expression was not affected. Furthermore, ARO treatment led to a decrease in anti-apoptotic mitochondrial Bcl-2 protein expression. On the other hand, pro-apoptotic mitochondrial Bax expression was not affected. However, the calculated Bax/Bcl-2 ratio (**Figure 11C**) showed an increase (up to 90% for MCF-7, 110% for MDA-MB-231) in favor of the pro-apoptotic proteins supporting a decreasing survival rate.

### 3.10 2D and 3D *in vitro* analyses of treatment with aronia combined with epirubicin in MCF-7 and MDA-MB-231 cells

The potential cytotoxic effects of combinatory co-treatment in MCF-7 and MDA-MB-231 cells were also analyzed. The nine mutual combinations of ARO and EPI were used in 2D and 3D models (**Table 8**). After a 72 h incubation in the 2D model, the best combinations (excluding A50/E50) were A50/E25, A25/E50 (MCF-7, MDA-MB-231) and A50/E12.5, A25/E25 (MDA-MB-231). In the 3D spheroids model, the best combinations were A50/E12.5, A50/E25, A25/E25, and A25/E50 in both tested cell lines. The combination A25/E25 showed inhibition similar to or better than a single IC_50_ EPI treatment (2D: MCF-7 = 0.54, MDA-MB-231 = 0.49; 3D: MCF-7 = 0.67, MDA-MB-231 = 0.65). Analyses also showed synergistic effect between ARO and EPI treatment (**Table 8**; **Supplementary Figure S1**): A25/E12.5, A25/E25, A12.5/E12.5, A12.5/E25 (MCF-7, 2D); A25/E12.5 (MDA-MB-231, 2D); A12.5/E25 (MCF-7, 3D); A50/E12.5, A50/E25, A25/E12.5, A12.5/E12.5, A12.5/E25, A12.5/E50 (MDA-MB-231, 3D).

**Table 8:** Resazurin metabolic test of ARO and EPI combinational treatment.

	Mutual combinations								
**2D**	A50/E12.5	A50/E25	A50/E50	A25/E12.5	A25/E25	A25/E50	A12.5/E12.5	A12.5/E25	A12.5/E50
MCF-7	0.53 ± 0.02	0.45 ± 0.02•	0.42 ± 0.03•	0.64 ± 0.03 **S**	0.52 ± 0.03 **S**	0.48 ± 0.02•	0.76 ± 0.04 **S**	0.72 ± 0.04 **S**	0.56 ± 0.04
MDA-MB-231	0.43 ± 0.01•	0.38 ± 0.02•	0.36 ± 0.03•	0.50 ± 0.02 **S**	0.44 ± 0.03•	0.42 ± 0.03•	0.65 ± 0.02	0.56 ± 0.02	0.51 ± 0.04
**3D**	A50/E12.5	A50/E25	A50/E50	A25/E12.5	A25/E25	A25/E50	A12.5/E12.5	A12.5/E25	A12.5/E50
MCF-7	0.52 ± 0.03•	0.49 ± 0.02•	0.48 ± 0.03•	0.60 ± 0.04	0.55 ± 0.05•	0.50 ± 0.02•	0.70 ± 0.04	0.64 ± 0.02 **S**	0.63 ± 0.02
MDA-MB-231	0.30 ± 0.03• **S**	0.26 ± 0.02•• **S**	0.25 ± 0.02••	0.48 ± 0.04 **S**	0.41 ± 0.02•	0.40 ± 0.03•	0.56 ± 0.01 **S**	0.56 ± 0.04 **S**	0.53 ± 0.05 **S**

Significant difference, • p > 0.05, ••p > 0.01vs IC_50_ of both ARO (A) and EPI (E) single treatments (**Table 7**). S, synergism. Data are presented as fold of untreated control.

## 4 Discussion

Current oncology research has demonstrated the significant anticancer activities of isolated phytochemicals and their mixtures present in whole plant foods (20, 35–38). BC is currently the tumor disease diagnosed most frequently worldwide in women (1). Extensive epidemiological studies show a reduced risk of BC associated with an increased intake of whole plant foods, including fruits, vegetables, and herbs (39–42). In this context, current oncology research emphasizes the importance of phytochemicals in reducing cancer risk through chemoprevention (7).

Phytochemicals, their derivatives or whole plant foods, and medicinal plants also represent promising possibilities for improving the effectiveness of conventional treatment in BC patients or suppressing adverse reactions induced by conventional therapies (43). In addition, the acquired resistance of cancer cells to chemotherapeutics represents a severe clinical problem for cytotoxic antitumor therapies [5]. Targeting the molecular pathways by using phytochemicals to improve therapeutic outcomes by increasing the sensitivity of cancer cells and reversing resistance to currently used therapeutic methods represents an essential preclinical and clinical research approach for improving the management of BC (5). The results of experimental studies have shown a direct connection between the administration of phytochemicals and the suppression or slowing of cancer development. Mechanisms of this effect include modulation of the cellular signaling pathways associated with key events for the initiation, promotion, and progression of BC, including oxidative damage, apoptosis, proliferation, angiogenesis, the formation of distant metastases, or the activity of tumor stem cells (35, 36, 44–46). Animal models of experimental BC represent a logical way of investigating the antitumor effect of phytosubstances in the multistep process of mammary gland carcinogenesis (17–24). Having proposed this idea, we assessed the anticancer potential of *A. melanocarpa* in therapeutic and preventive rodent BC models and human BC cells *in vitro*. In our study, the mechanism of action of aronia was analyzed by histopathological, immunohistochemical, and molecular-biological methods, which represent a highly valid approach to evaluating tumorigenesis. The dosing of phytosubstances in this study was optimized according to our previous experience with given animal models. Rodents generally show different pharmacokinetics and pharmacodynamics of many phytosubstances compared to humans.

For this reason, higher doses of the whole plants tested are used in experimental studies to verify the antitumor effects of phytosubstances (17). The content compounds of aronia were analyzed using LC-MS. This analysis confirmed the presence of anthocyanins in aronia. Anthocyanins had been reported previously in aronia (47). They are described as antioxidant and antiradical compounds, and the effects, revealed also in our cellular antioxidant assay, are by previously published studies (9). The content of anthocyanins may also be influenced by the effects observed in our assays on the MCF-7 and MDA-MB-231 cell lines. Anthocyanins are described as compounds interfering with inflammatory pathways (48). They decrease epithelial to mesenchymal transition *via* Sirt1 promotion and NF-κB inhibition. In addition, they down-regulate the expression of matrix metalloproteinases, which can decrease tumor invasiveness and the potential risk of forming metastasis (49). They interact with estrogenic receptors (50, 51). Furthermore, they possess antiangiogenic effects by effects on VEGF (52). They decrease EGRF/Akt signaling and increase mir-124 expression (52, 53). The effects of different anthocyanins on many other cellular targets were previously summarized by Li et al. (54).

The therapeutic mouse 4T1 model is characterized by high tumorigenicity and invasiveness with the formation of spontaneous metastases from the primary tumor to several distant regions, thus imitating the course of an aggressive form of mammary gland carcinogenesis in humans. In our evaluation of the therapeutic effects of aronia in the syngeneic 4T1 model of mammary carcinogenesis, we specifically focused on tumor volume as a fundamental parameter of mammary carcinogenesis (tumor staging). Additionally, we examined the histopathological assessment of the mitotic index and the proportion of necrosis in the tumor tissue (tumor grading). The results showed that aronia significantly reduced tumor volume in both treated groups compared to the control group. Furthermore, aronia, at both concentrations, demonstrated a dose-dependent reduction in the mitotic activity index compared to the control group. These findings align with the previous research conducted by our working group, where cinnamon (Cinnamomum zeylanicum L.) exhibited similar antitumor effects, including a significant reduction in the volume of 4T1 tumors and a dose-dependent decrease in the mitotic activity index (23). Thyme (*Thymus vulgaris* L.) significantly reduced the volume of 4T1 tumors in both doses compared to the control group; in addition, thyme significantly reduced the index of mitotic activity and reduced the necrosis/total tumor tissue ratio (22). The current results of our working group have revealed significant beneficial efficacy of *Rhus coriaria* L (24). and *Salvia officinalis* L (55). in the same 4T1 model of BC. We can conclude that the current and previous results of our working group confirm the apparent antitumor efficacy of phytosubstances comparable to the therapeutic effects of synthetic drugs evaluated in the same mouse 4T1 model (56–58). Despite the proven antitumor effects of aronia and other plant foods in our laboratory using the 4T1 therapeutic mouse BC model, their potential use in the therapy of human BC requires further intensive investigation at the preclinical and clinical levels. The 4T1 model has several important limitations; for example, t uses only one tumor cell line and has a high degree of uniformity in experimental conditions that cannot be reached in a clinical setting.

The chemopreventive effects of aronia were assessed by examining various parameters of carcinogenesis, such as tumor frequency, incidence, latency, and volume. Additionally, histopathological evaluations of mammary lesions and the ratio of poorly and well-differentiated (high/low grade) carcinomas were conducted. Aronia demonstrated a noticeable, albeit statistically insignificant, decrease in the ratio of poorly and well-differentiated (high/low grade) carcinomas by 34% at the lower dosage and 38.5% at the higher dosage compared to the control group. Furthermore, a slight reduction in tumor volume and an extension of tumor latency were observed following treatment with aronia at the higher dosage compared to the control group. These findings align with our previous robust research using the same chemically induced rat mammary carcinogenesis model. In these studies, chlorella, a blend of dark fruit peels, oregano, cloves, thyme, cinnamon, and sumac, exhibited significant decreases in incidence, frequency, and volume values, as well as an elongation of latency and a reduction in the ratio of poorly and well-differentiated (high/low grade) carcinomas (17, 19–24). Other researchers have also reported significant chemopreventive effects of plant-based foods on the development of mammary carcinogenesis *in vivo*. Blueberries and blackberries have shown chemopreventive and therapeutic activity *in vivo* by reducing tumor volume, proliferation, and lengthening latency (25, 26). In another study, rosemary significantly reduced the tumor frequency in DMBA-induced rat mammary carcinogenesis, demonstrating its significant chemopreventive activities *in vivo* (59). Finally, pomegranate exerted chemopreventive efficacy in carcinogen-induced rat mammary tumorigenesis by proapoptotic and antiproliferative mechanisms of action (60).

Recent research indicates that phytochemicals and their natural combinations play a significant role in regulating the processes involved in cancer development, such as apoptosis, proliferation, angiogenesis, and mechanisms related to cancer stem cells (20). Our study focused on examining the anti-tumor effects of entire plants in a chemopreventive setting by analyzing selected markers of these mechanisms through immunohistochemical and molecular techniques.

Programmed cell death is an essential process for the optimal functioning of an organism. Apoptosis can be triggered by either an intrinsic or extrinsic apoptotic pathway. In our research, we examined the impact of aronia on the internal signaling of programmed cell death, specifically through the mitochondria. The activation of caspases plays a pivotal role in apoptosis, with caspase-3 being recognized as an effector caspase involved in the initiation of programmed cell death. Additionally, proteins such as Bax, which promotes apoptosis, and Bcl-2, which inhibits apoptosis, also regulate this process (18). Intracellular responses to various stress signals, such as oxidative stress, DNA damage, and hypoxia, initiate the intrinsic apoptotic pathway. These responses result in the activation of pro-apoptotic proteins and the downregulation of anti-apoptotic proteins. As a result, the permeability of the outer membrane of the mitochondria is disrupted, leading to the release of specific proteins, including cytochrome c, into the cytosol. Cytochrome c, when bound to other proteins, triggers the formation of a complex known as the apoptosome. This complex then activates caspase 9, which subsequently activates caspase 3. Activating caspase 3 cleavages key cell substrates, ultimately resulting in cell death (61). The dysregulation of apoptosis, an intricately orchestrated process, stands as a fundamental mechanism underlying the progression of tumors (35). Phytochemicals play a crucial role in regulating the process of tumor cell apoptosis, augmenting the Bax/Bcl-2 ratio results in heightened caspase-3 activity, ultimately triggering the initiation of cancer cell apoptosis (18). In this study, we evaluated modifications in the expression of three parameters of apoptosis, namely cytoplasmic caspase-3, Bax, and Bcl-2.

At a higher dose, aronia significantly increased the cytoplasmic expression of caspase-3 compared to the control group. Aronia administration at both doses significantly increased Bax expression by over 90% compared to the control group. Additionally, a notable decrease in Bcl-2 expression was observed compared to controls. This substantially increased the Bax/Bcl-2 expression ratio by 173% (ARO 0.3) and 142.5% (ARO 3) versus the control group. Consistent results were also obtained from *in vitro* experiments. Furthermore, *in vitro* findings demonstrated a decrease in mitochondrial membrane potential, cell cycle arrest in the G1 phase linked to reduced phospho-Rb checkpoint protein expression, and the initiation of apoptosis following aronia treatment. These effects of aronia may be attributed to its high anthocyanin content, which has previously exhibited pro-apoptotic properties (54).

Previous studies conducted in our laboratory have also demonstrated the pro-apoptotic effect of various whole plants. These studies revealed a significant correlation between an increase in the expression of caspase-3 and an increase in the ratio of Bax/Bcl-2 in BC cells *in vivo*. This correlation was observed after administering dark fruit peels, oregano, cloves, cinnamon, sumac, and salvia in the NMU-induced chemopreventive model of mammary carcinogenesis in rats (19–21, 23, 24, 55). The findings from our experiments indicate that specific plant foods can potentially induce cancer apoptosis in BC effectively.

Except for apoptosis, cancer cells are characterized by altered signaling, leading to excessive proliferation and the circumvention of signals that in normal cells precisely regulate the cell cycle (62). Naturally occurring phytosubstances can directly regulate the proliferation of estrogen-dependent as well as estrogen-independent mammary tumor cells by influencing essential mechanisms such as the inhibition of COX-2 or the modulation of key signaling pathways (Hedgehog, NF-κB, Nrf2, STAT3, Wnt and others) (63–65). The nuclear protein Ki67 is a good marker of tumor cell proliferation because it is associated with active phases of the cell cycle, whereas it is absent from resting stages (66). Evaluation of tumor cell proliferation in this study showed a dose-independent non-significant reduction of Ki67 expression by 9% and 5% in the aronia groups compared to the control. On the other hand, in our previous chemopreventive studies, we observed a significant reduction of Ki67 caused by a mixture of dark fruit peels, young barley leaves, oregano, cloves, cinnamon, and sumac (18–21, 23, 24). Similar preclinical *in vivo* data showing the antiproliferative effects of plant foods have also been published by other authors who tested bilberries (67) and *Trianthema portulacastrum* L (68). A clinical study analyzing the effect of green tea on the expression of Ki67 in BC demonstrated a significant reduction in benign and non-significant decreases in malignant tissues of patients with DCIS or stage I/II BC (69).

Tumor neovascularization is necessary for the progression of cancer disease and the spread of metastases (45). Vascular endothelial growth factor A (VEGF-A) and its receptors (VEGFR) are important for physiological and pathological angiogenesis processes. Although the VEGF-A binds to both its receptors (VEGFR-1, VEGFR-2), the primary signaling of endothelial cell proliferation and vascular permeability is mediated by the binding to VEGFR-2, and that is why VEGFA/VEGFR-2 represents the most important ligand-receptor complex necessary for tumor angiogenesis (70). In this study, aronia dose-independently decreased the expression of VEGF by 39.5% and 37%, compared to the control group. Similarly, as we described in previous chemopreventive animal studies using dark fruit peels, oregano, cloves, thyme, and cinnamon, we have demonstrated their significant anti-angiogenic potential *via* the downregulation of VEGF/VEGFR-2 expression (19–23). Natural mixtures of plant secondary metabolites have also shown significant antiangiogenic effects in other experimental BC models (71–73), warranting comprehensive preclinical and clinical analyses on this research topic.

The antitumor effect of phytochemicals also includes significant action on the vitality of cancer stem cells (23, 24, 36, 74, 75). Cancer stem cells represent a subpopulation characterized by the possibility of self-renewal and differentiation into different cell types. Cancer stem cells are involved mainly in the multi-step process of carcinogenesis, including tumor initiation, promotion, progression, the formation of metastases, or later resistance to therapy (76). Within the clinical practice and oncology research, several clinically established cancer stem cell markers exist, including CD24, CD44, CD133, ALDH1, and EpCAM (77, 78). In this study, aronia at a higher dose noticeably reduced the expression of CD133 by 58.5% compared to the control group. In our recent experiments using rat mammary carcinomas *in vivo*, we demonstrated the significant effects of sumac and salvia on CSC markers (24, 55). We found a dose-dependent significant decrease in CD24, ALDH1, and EpCam expression after the sumac treatment. Sage reduced the expressions of ALDH1 and EpCam in rat BC cells. Using the same animal model, we demonstrated a significant effect of oregano, cloves, thyme, and cinnamon on various cancer stem cell parameters (20–23). Preclinical studies pointed to higher antitumor efficacy of whole plants containing mixtures of bioactive phytochemicals (*via* the modulation of multiple cell signaling pathways and mechanisms, including CSCs) as compared to isolated phytochemicals (36, 37). Data from our laboratory and other authors (79–81) call attention to the need for in-depth research in this area and its consequent translation to clinical oncology.

In recent years, cancer prevention and therapy research has been concentrating on the impact of phytochemicals on specific carcinogenesis processes, particularly the modulation of epigenetic mechanisms. The investigation of abnormal epigenetic mechanisms has garnered significant interest in the BC field due to the reversible nature of epigenetic changes, which occur early in cancer development. Epigenetic mechanisms, such as histone post-translational modifications, specific miRNA expressions, and gene promoter methylation, can influence cellular processes. These mechanisms can affect cell cycle regulators, nuclear receptors, tumor-suppressor genes, transcription factors, and gene products involved in apoptosis or repair mechanisms, ultimately playing a role in cancer development (82–84).

Post-translational modifications of histones and their possible aberrations were an important epigenetic parameter in this experiment with aronia in an animal model of BC. Aronia beneficially decreased H3K4m3 and H3K9m3 levels in cancer cells compared to controls. In addition, apparent but not significant increases in H4K16ac and H4K20m3 were observed in treated groups vs controls. In our recent study, salvia significantly reduced H3K4m3 levels and increased H4K16ac levels in rat BC samples (55). In the same rat BC model, we described positive alterations in post-translation modifications of histone molecules in BC cells *in vivo* after chemoprevention with clove buds (21), thyme (22), cinnamon (23), and sumac (24). Several researchers have noted comparable epigenetic alterations involving resveratrol, leading to the reactivation of tumor-suppressor genes BRCA1, TP53, and TP21 in MCF-7 BC cells. The hindrance of cancer advancement was linked to a reduction in specific post-translational modifications of histones, such as a decrease in dimethylation or trimethylation of histones H3 and H4 (H4R3m2, H3K27m3), and an elevation in the levels of acetylation of histones H3 (H3K9ac, H3K27ac). These modifications were correlated with the reactivation of the aforementioned tumor-suppressor genes (85). 1. Additionally, the co-administration of sulforaphane and withaferin reduced HDAC enzyme activity, leading to a decrease in acetylation levels. This disruption in histone chemical modifications ultimately caused a decrease in cancer cell viability and triggered programmed cell death in MCF-7 and MDA-MB-231 cells (86). Furthermore, the identical blend of phytochemicals elevated the histone methylation levels, inhibiting the cell cycle in MCF-7 and MDA-MB-231 cells (87).

In general, miRNAs play a crucial role in regulating gene expression and thus can significantly modulate processes of carcinogenesis (88). Specific miRNAs have been introduced as clinical markers in the diagnosis, prognosis, and prediction of therapy for BC. In this regard, we have used in this study well-validated oncogenic miRNAs - miR21, miR155, and miR210 and tumor-suppressive miRNAs - miR22, miR34a, and miR145 as negative/positive regulators of mammary carcinogenesis (89, 90). Some phytochemicals have been documented as efficient tools that affect miRNA expression (91). In this study, aronia showed significant positive regulatory effects using the above-mentioned miRNA markers. Aronia downregulated oncogenic miR155 and upregulated tumor-suppressive miR145 compared to control rat BC samples. An apparent tendency towards the dose-dependent upregulation of miR22 expression was found after treatment with aronia. Oncogenic miR-210 significantly affects cancer by modulating the hypoxia-inducible factor (HIF) signaling (92). On the other hand, miR210 is manifested as a tumor suppressor in normoxic conditions, i.e., during the initiation of cancer (22, 93). In this study, aronia in both doses significantly increased miR210 expression in rat BC specimens compared to controls. We can hypothesize that miR210 behaved as a tumor-suppressor in our aronia study due to the initial stages of the rat BC tumors evaluated (we found only a small average tumor volume < 1 cm^3^). Significant regulatory effects of the plant nutraceuticals *T. vulgaris, C. zeylanicum*, and *R. coriaria* on miRNA expressions were observed in our previous rat chemoprevention studies using the same model (22–24). Anticancer effects of plant-derived molecules on breast carcinogenesis through the modulation of cancer-associated microRNAs have also been observed in other laboratories (94, 95).

The hypermethylation/hypomethylation of promoter regions of tumor-suppressor genes (TSG) significantly affects processes of carcinogenesis (96). This study analyzed the methylation status of standardized CpG islands of the TSG promoters ATM, PITX2, RASSF1A, PTEN, and TIMP3. These represent clinically well-validated TSG, frequently downregulated in BC patients (97) or in animal models (98) due to promoter hypermethylation. This study revealed that high-dose treatment with aronia significantly reduced the methylation status in the *TIMP3* promoter and non-significantly decreased the promoter`s methylation by 11% in both the *ATM* and *RASSF1* genes. However, at both doses, aronia markedly increased the methylation of the *PTEN* promoter by approx. 150% vs. control samples. Recent data from our laboratory using the same chemoprevention model *in vivo* documented unambiguous significant hypomethylating effects of natural phytochemical mixtures on the specific TSG promoters´ methylation status after treatment with *Syzygium aromaticum* L., *T. vulgaris*, *C. zeylanicum*, *R. coriaria, and S. officinalis* (21–24). Demethylation effects of plant nutraceuticals on TSG promoters’ regions have also been described in the cancer models also by other laboratories (99, 100). Despite growing research data, there is still insufficient knowledge of the role of phytochemicals on epigenetic changes in cancer. In addition, most of these data are preclinical and cannot be easily translated into clinical oncology. Future research must resolve several needs in the epigenomics of cancer: (a) more detailed insight into the molecular mechanisms and targets of phytochemicals, (b) a definition of effective (individual) dosing, (c) the evaluation of the combined therapy of plant bioactive agents with conventional chemotherapeutics to target relevant epigenetic mechanisms, (d) innovative methods to improve the bioavailability of phytochemicals in the recipient organisms, and (e) the individual characteristics of the cancer patient (7).

Phytochemicals could also contribute to traditional chemotherapy as cofactors to reduce the dosage of chemotherapy and thus also the unwanted side effects or the sensitizing of chemoresistant cancer cells. The beneficial effects of combination treatment are already widely accepted as part of new strategies in cancer research, as has been reviewed (101, 102). Our preliminary *in vitro* data showed a beneficial effect of such combinations where IC_25_ concentrations of aronia and epirubicin showed inhibitory effects similar to or increased as those of a single epirubicin treatment. In addition, several combinations showed synergistic effects. Despite these promising results, further research is needed to optimize the combinational/re-sensitizing therapeutic approach in clinical oncology.

## 5 Conclusion and future perspectives

Current global trends in BC incidence and mortality require the introduction of more effective approaches to managing this disease. To ensure effective health care for BC patients, modern medicine requires an individual approach and the identification of modern and more effective therapeutic strategies, including increased sensitivity of cancer cells to conventional chemotherapeutics. Plant nutraceuticals are a rich source of bioactive phytochemicals with significant effects on human health, characterized by easy availability and a toxicological profile better than chemotherapeutic drugs. Phytosubstances can demonstrably regulate the multistep process of mammary carcinogenesis via the modulation of numerous cell signalings involved in carcinogenesis. Based on the above discussion, phytochemicals represent potentially effective substances for use in new treatment strategies and the prevention of BC. The incidence of BC can be reduced by timely choice of the right and prevention.

*A. melanocarpa* exhibited significant antitumor effects in a therapeutic model of 4T1 mouse adenocarcinoma. This was observed through a notable decrease in tumor volume and the mitotic activity index of cancer cells. Additionally, aronia displayed significant antitumor activity in a chemopreventive model of experimental BC in female rats and a model of human BC adenocarcinoma using MCF-7 and MDA-MB-231 cells. This activity was attributed to its proapoptotic, antiangiogenic, antiproliferative, and anti-CSC effects and positive epigenetic changes. Our findings highlight the activation of non-specific signaling pathways in cancer cells, which contribute to the anticancer efficacy of aronia across three distinct BC models. Therefore, *in vitro* and *in vivo* experimental BC models are crucial in evaluating novel therapeutic and chemopreventive agents. However, introducing phytochemicals in routine oncological practice requires comprehensive in-depth clinical research, which must precisely define all their effects on the organism, including mechanisms and correct dosage in oncology patients. Using plant nutraceuticals in the clinical management of BC requires comprehensive clinical analyses, which must resolve several key issues within oncology practice:

1. defining pharmacokinetics/dynamics that can determine the effective and safe dosing and modes of administration;.
2. determination of sensitive cancer subtypes respecting the individual characteristics of patients;.
3. it is essential to outline appropriate uses of plant nutraceuticals in conjunction with traditional medications to enhance the re-sensitization of cancer cells, inhibit metastatic growth, and boost the immune system in patients.

The intricate pathways of cellular, subcellular, and molecular mechanisms involved in carcinogenesis are expressed at a comprehensive multi-omics level. As a result, these pathways are regarded as crucial targets for advanced diagnostics and cost-effective management of BC, encompassing therapy and preventive measures (8). 1. The extensively documented anti-cancer properties of phytochemicals and plant nutraceuticals are crucial for enhancing patient outcomes and have the potential to be utilized in primary (preventing cancer development), secondary (preventing possible spread to other parts of the body), and tertiary (preventing additional complications) care within clinical settings (8). In this regard, precise data analyses are crucial to adapting BC management`s algorithms to individual patient profiles. The rapidly growing incidence of BC in the general population and, in particular, the aggressive metastatic BC sub-types found in the young female population prompt clinicians to apply advanced screening methods and targeted individualized and preventive approaches to overall BC management (103). Predictive diagnostics, patient stratification, targeted prevention, and treatments focused on the individualized patient multiomics characteristics represent effective tools for the cost-effective application of plant nutraceuticals to optimize BC management. The primary, secondary, and tertiary approach in BC management within the framework of predictive, preventive, and personalized medicine is considered an advanced strategy to improve both individual outcomes and those of society at large (104–108).

## Data Availability

The original contributions presented in the study are included in the article/**Supplementary Material**. Further inquiries can be directed to the corresponding authors.
