# Supplementary figures and images for: Aronia melanocarpa L. fruit peels show anti-cancer effects in preclinical models of breast carcinoma: The perspectives in the chemoprevention and therapy modulation
Source: Front Oncol. 2024 Oct 7;14:1463656. doi: 10.3389/fonc.2024.1463656 (PMC11491292; doi:10.3389/fonc.2024.1463656)

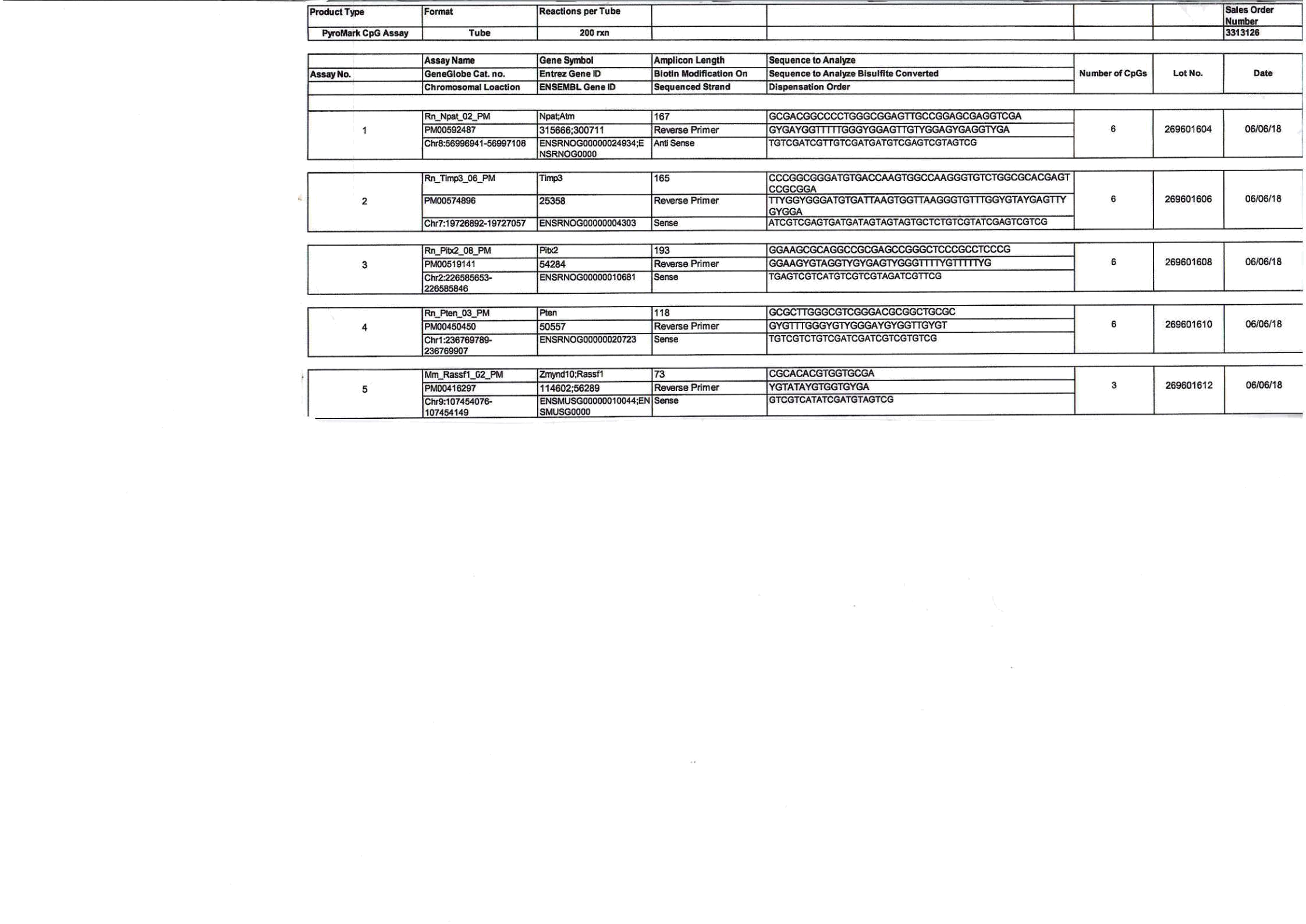
Table S1. Primer sequence

Figure S1. Isobolograms of ARO and EPI combinations


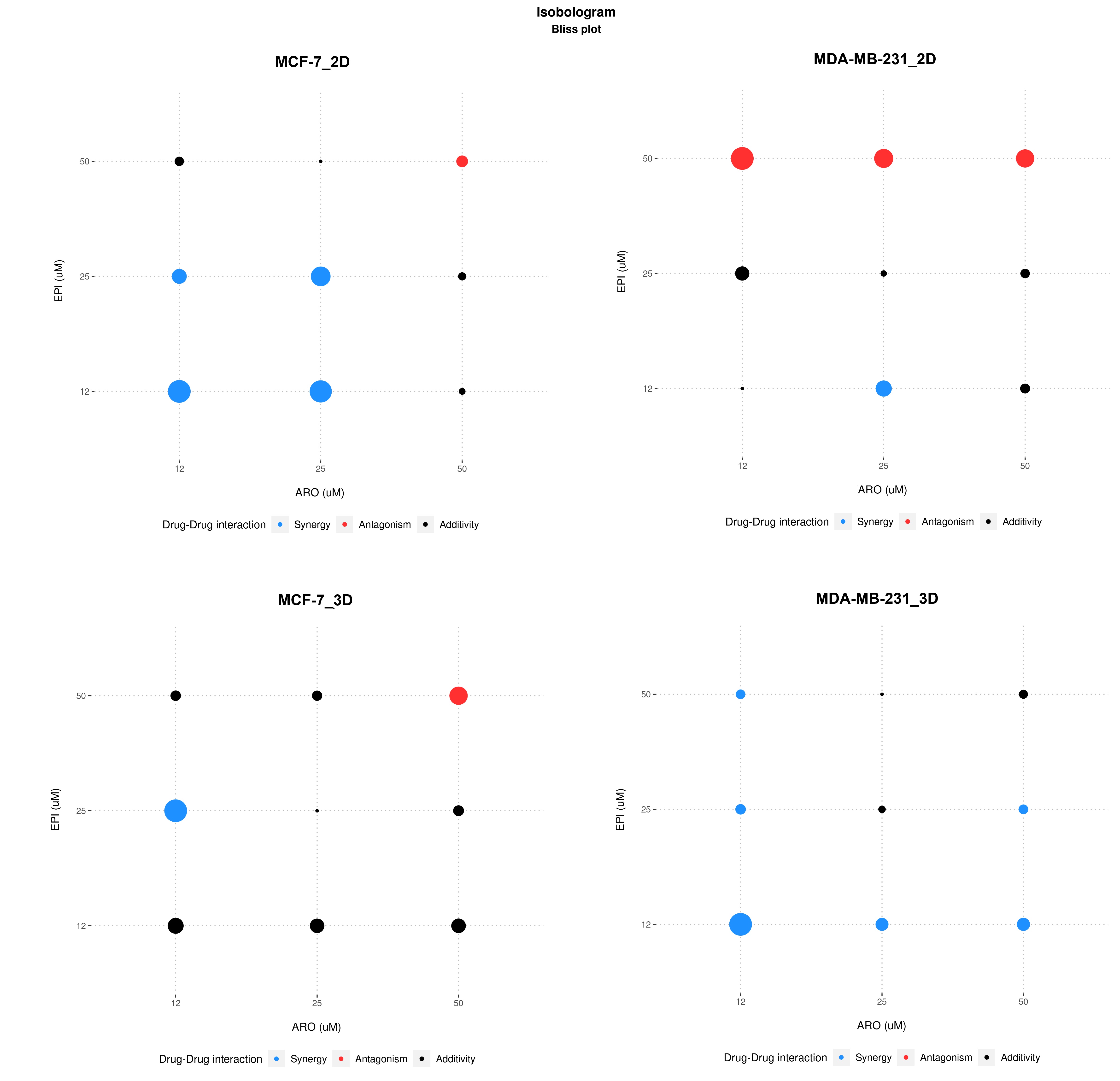

Supplement: Supplementary file 1 [file DataSheet1.doc]
